# Numerical model of a top-blown rotary converter preheating and charge heating with an oxy-fuel burner

**DOI:** 10.12688/openreseurope.18594.2

**Published:** 2025-07-04

**Authors:** Sergey Semenov, Patrick Namy, Aditya Kale, Sello Tsebe

**Affiliations:** 1 SIMTEC, Grenoble, 38000, France; 2 MINTEK, Randburg, 2194, South Africa

**Keywords:** Top-Blown Rotary Converter, Furnace Preheating, Charge Heating, Aluminothermic Reduction, Finite Element Method, COMSOL Multiphysics, Heat Transfer Modelling, Phase Change

## Abstract

**Background:**

The present work is conducted in the framework of the SisAl Pilot EU project, which aims to optimize silicon production in Europe by recycling materials and using carbon-emission-friendly technology. Silicon production experiments were conducted on laboratory and pilot scales in different types of furnaces, including top-blown rotary converters (TBRC) used as chemical reactors for molten slag-metal mixtures. In addition to experimental work, process optimization also relies on numerical modelling.

**Methods:**

In this study, COMSOL Multiphysics® was used for the numerical testing of a new thermal design of TBRC by simulating its preheating and charge heating owing to an external heat source provided by an oxy-fuel burner.

**Results and conclusions:**

The risk of slag solidification in TBRC during the aluminothermic reduction of silica was assessed. The model predicts that, with a useful burner power of 600 kW, the empty TBRC can be preheated to 1650°C in less than 30 min. Based on this model, the optimum burner power for maintaining the TBRC charge in a liquid state was determined. The influence of the TBRC inclination angle and its rotation frequency was studied numerically.

## 1. Introduction

This work was conducted in the framework of the SisAl Pilot EU project, which is focused on demonstrating the possibility of metallurgical-grade silicon production at the pilot scale based on the aluminothermic reduction of silica. The traditional carbothermic reduction of silica ^1– 3^ in a submerged arc furnace (SAF) involves multiple chemical reactions in all phases (solid, liquid and gaseous). In an idealized form, the overall reduction process can be represented by the following chemical reaction ^1, 3^:

$${\text{SiO}}_{\text{2}}+2\;\text{C}⇌\;\text{Si}+\;2\;\text{CO}$$

In comparison with the traditional carbothermic reduction of silica ^1– 3^, the advantage of the proposed aluminothermic reduction technology is its low CO2 emissions and the possibility to recycle generated waste back into the process ^16^. Additionally, the exothermic nature of the aluminothermic reaction provides energy efficiency and lowers the required temperature ^4^. The main chemical reaction involved is shown below ^4^:

$$4\;\text{Al}+3\;\;\text{Si}{\text{O}}_{2}\;\left(\text{in}\;\text{CaO}–\text{Si}{\text{O}}_{2}\;\text{slag}\right)\;\;\;⇌\;\;3\;\text{Si}+2\;\text{A}{\text{l}}_{2}{\text{O}}_{3}\;\left(\text{in}\;\text{CaO}–\text{A}{\text{l}}_{2}{\text{O}}_{3}\;\text{slag}\right)$$

A preliminary economic assessment of the SisAl process is considered in 5. Unlike the gas-solid reactions in the conventional carbothermic process, the SisAl process operates through liquid-liquid reactions. This eliminates the need for specific mechanical strength and particle size requirements for the quartz material. As a result, the SisAl process can utilize less commonly used and more cost-effective silica (SiO _2_) sources ^5^. Relatively expensive quicklime (CaO) can also be replaced by cheaper secondary materials from the mining or metallurgical industries ^5^. Aluminum is the most expensive raw material in the SisAl process, making white dross a cost-effective alternative for pilot testing ^5^.

As part of the SisAl Pilot project, the numerical modelling support of the experimental works was stipulated. One of these efforts is focused on developing a numerical model of a top-blown rotary converter (TBRC) heated by an oxy-fuel burner. Within the SisAl Pilot project, this metallurgical reactor for the aluminothermic reduction of silica was proposed by MINTEK as an alternative to an electric furnace to mitigate the risks associated with the interruption of the electrical power supply due to load shedding in South Africa. This type of reactor offers operational flexibility, enhanced mixing behavior, and potential for continuous processing. Unlike traditional converters or rotary kilns, the TBRC enables better control over temperature and chemical reactions through its unique combination of rotary motion and top-blown gas or flame injection.

Several literature sources report the results of numerical modelling of heat and mass transfer in rotary furnaces. Examples of numerical models of heat transfer in a rotary furnace heated by gas burners in the presence of charge can be found in 6 and 7. Gas flow models are reported in 8 and 9. A multiphase computational fluid dynamics model of charge stirring with oxygen jets and argon injection is presented in 10. Recent numerical work on a rotary furnace ^11^ models its preheating with a gas burner, as well as heat transfer and charge flow during furnace operation. Most existing literature focuses either on empirical or post-operational data. Limited work has been published on predictive numerical modeling to validate TBRC design performance prior to construction, especially within project-specific constraints like those of the SisAl Pilot project. This study addresses this research gap by conducting a numerical investigation aimed at validating a specific TBRC design before physical construction.

Key distinguishing aspects of our work compared to previous studies of rotary converters were the geometry of the problem, the refractory materials and the operating conditions, which directly influence the flow dynamics and heat transfer in TBRC. The objective of the present modelling work was to test the thermal performance of TBRC during the preheating and aluminothermic reduction stages. Molten slag (50wt%CaO-50wt%SiO _2_) and aluminum are planned to be prepared in separate furnaces outside the TBRC and then poured into the TBRC for aluminothermic reduction to take place. The risk of slag solidification in TBRC during the aluminothermic reduction stage needs to be assessed. The optimal burner power for maintaining the TBRC charge in the liquid state must be determined. The influence of the TBRC inclination angle and its rotation frequency should also be numerically studied. By aligning the modeling approach with the functional needs of the SisAl Pilot project, the study contributes practical insights toward design verification rather than theoretical advancement. In Section 2 and Section 3, the model geometry and numerical methods are described, respectively. Section 4 and Section 5 present the governing equations and material properties, respectively. In Section 6, numerical results are presented and discussed. Finally, Section 7 concludes the paper.

## 2. Problem geometry

**Figure 1.**
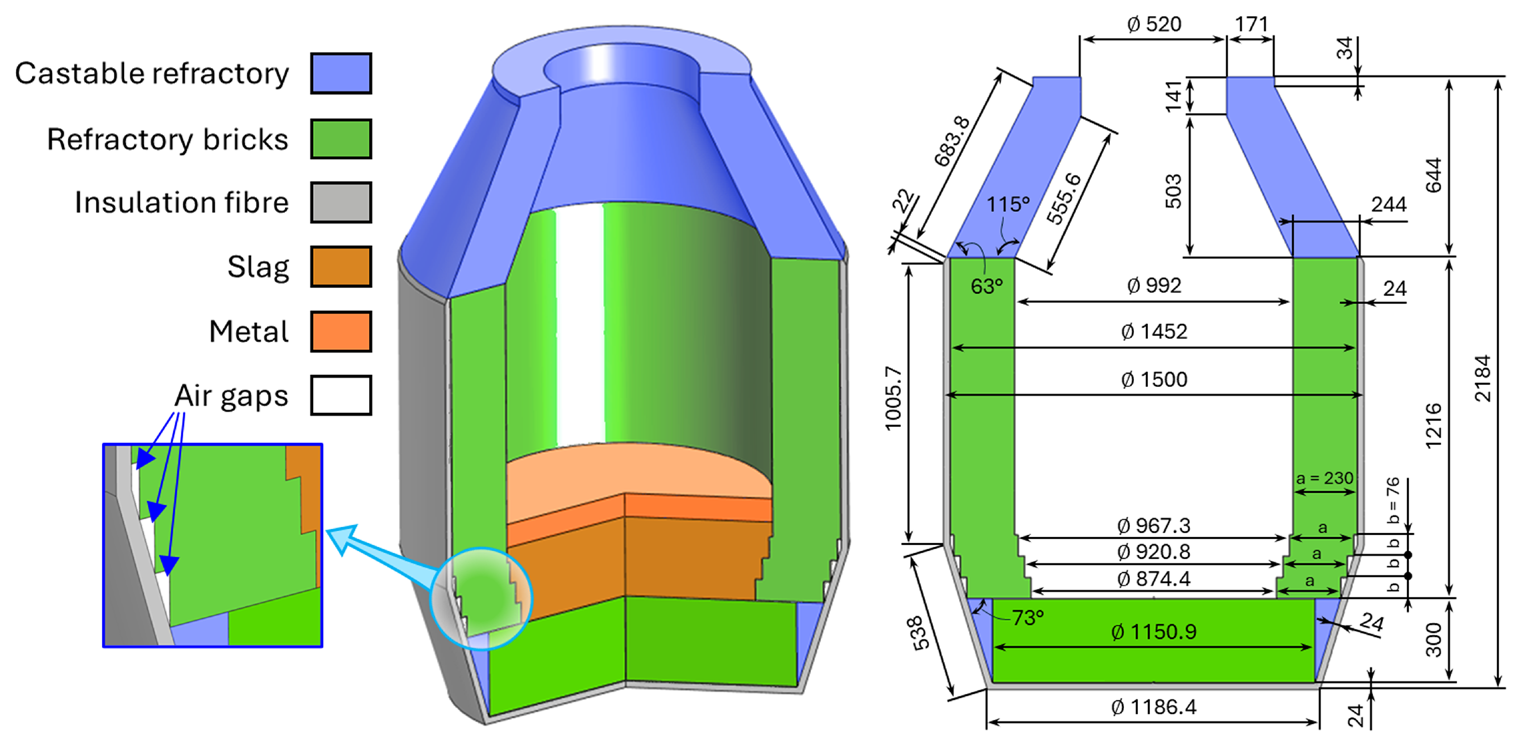
Modelled axisymmetric geometry of TBRC. Dimensions in mm.

The geometry and dimensions of the top-blown rotary converter (TBRC) were based on technical drawings provided by MINTEK. The corresponding numerically modelled axisymmetric geometry of the TBRC and materials are shown in Figure 1. The steel shell is not modelled because its geometry is not known prior to furnace construction, and because its contribution to the wall’s thermal resistance is insignificant owing to the high thermal conductivity of steel and the relatively small shell thickness. The heights *H _s_* and *H _m_* of the slag and metal layers inside the TBRC are modelled based on the materials’ initial volumes *V*_0, *s*_ and *V*_0, *m*_ and on the geometry of internal cavity of the TBRC, which depends on its spatial orientation.

**Figure 2.**
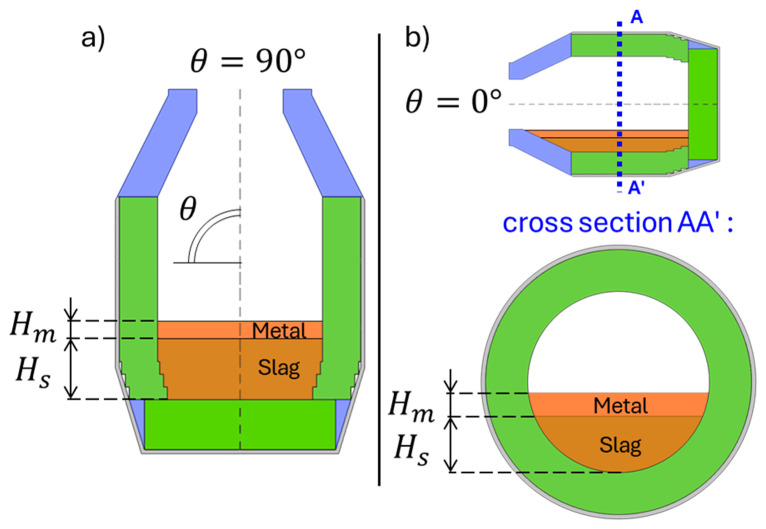
Numerically modelled orientations of TBRC. ( **a**): Vertical orientation. ( **b**): Horizontal orientation.

Two TBRC orientations were numerically modelled in this work: vertical orientation (see Figure 2 (a)) and hypothetical horizontal orientation (see Figure 2 (b)). The initial volumes *V*_0, *s*_ and *V*_0, *m*_ of slag and metal, respectively, are computed from their initial masses *m*_0, *s*_ = 500 kg and *m*_0, *m*_ = 150 kg and their initial densities *ρ*_0, *s*_( *T*_0, *s*_, **X**_0, *s*_) and *ρ*_0, *m*_( *T*_0, *m*_, **X**_0, *m*_) which depend on their initial temperatures *T*_0, *s*_ and *T*_0, *m*_ and initial compositions **X**_0, *s*_ and **X**_0, *m*_.

$$V_{0,k}=m_{0,k}/\rho_{0,k}(T_{0,k},{\text{X}}_{0,k}),\;k=s,m$$

$${\text{X}}_{0,s}=(X_{0,SiO_{2}},X_{0,Al_{2}O_{3}},X_{0,CaO})$$

$${\text{X}}_{0,m}=(X_{0,Si},X_{0,Al},X_{0,Ca})$$

where the subscripts *s* and *m* stand for slag and metal, respectively, *X*_0, *i*_ stands for the initial mole fraction of species *i*.

## 3. Methods

**Figure 3.**
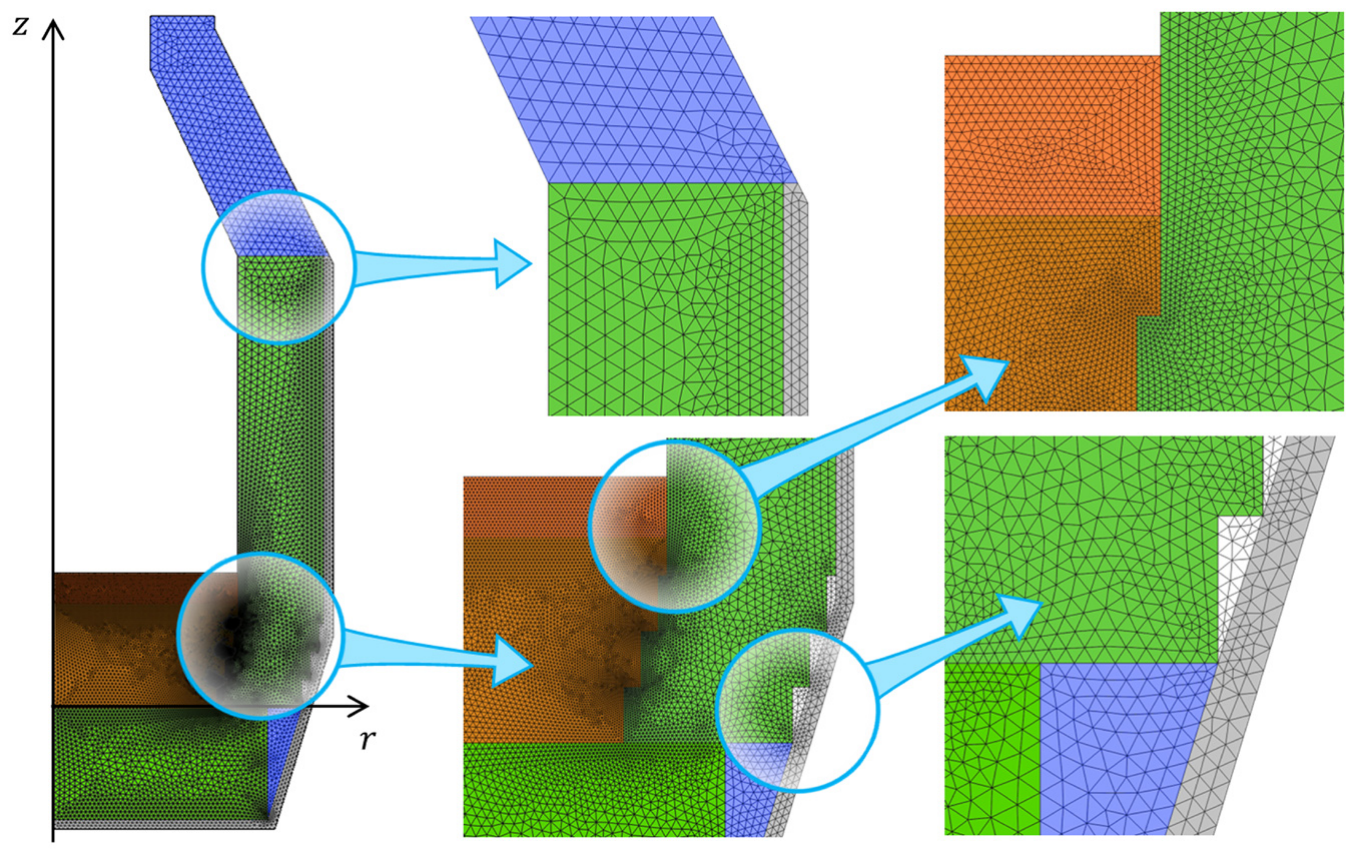
Computational mesh.

This problem was solved using the finite element software COMSOL Multiphysics ^®^ version 6.1 ( www.comsol.com, a copyright license is obtained). Modules of Heat Transfer, Turbulent Flow k–ω, and Surface-to-Surface Radiation were used to set up the model physics. The temperature and velocity fields were spatially discretized using quadratic elements, whereas the pressure and surface radiosity were discretized using linear elements. Time integration is performed with a Backward Differentiation Formula (BDF) of orders 1 to 2. The computational domain was spatially discretized with a triangular mesh which consists of 27 600 finite elements (see Figure 3), resulting in 220 700 degrees of freedom. The computations were performed on two laptops with eight physical cores, an Intel processor with 64 GB RAM, and a workstation with 64 physical cores and 1 TB RAM.

## 4. Governing equations

The physics of the problem includes heat transfer in solids and fluids with phase change, surface-to-surface radiation, and a turbulent k–ω model to simulate slag and metal flow. All the transport equations and boundary conditions were similar for both the vertical and horizontal orientations of the TBRC. Without a loss of generality, only the vertical configuration is presented in this section.

### 4.1. Fluid dynamics in metal phase

Unsteady incompressible RANS equations with *k-ω* turbulence model are solved in the metal domain:

ρ(∂u/∂t+u⋅∇u)=∇⋅[−pI+K]+ρg

∇⋅u=0

$$\rho (\partial k/\partial t+\text{u}\cdot \nabla k)=\nabla \cdot [\mu_{k}\nabla k]+P_{k}-\beta_{0}^{*}\rho \omega k$$

$$\rho (\frac{\partial \omega }{\partial t}+\text{u}\cdot \nabla \omega )=\nabla \cdot [\mu_{\omega }\nabla \omega ]+\text{α}\frac{\omega }{k}P_{k}-\beta_{0}\rho \omega^{2}$$

$$\text{K}=(\mu +\mu_{T})(\nabla \text{u}+{\left(\nabla \text{u}\right)}^{\text{⊤}}),\;\mu_{T}=\rho k/\omega ,\;\mu_{k}=\mu +\mu_{T}\sigma_{k}^{*},\;\mu_{\omega }=\mu +\mu_{T}\sigma_{\omega }$$

$$P_{k}=\mu_{\text{T}}\left[\nabla \text{u}\text{:}(\nabla \text{u}+(\nabla \text{u}{)}^{\text{⊤}})\right],\;\alpha \text{=}0.52,\;\sigma_{k}^{*}=0.5,\;\sigma_{\omega }=0.5,\;\beta_{0}=0.072,\;\beta_{0}^{*}=0.09$$

In this model, the turbulent viscosity *μ _T_* was calculated from two additional fields: the turbulent kinetic energy *k* and the specific rate of dissipation of the turbulent kinetic energy *ω*. The axial symmetry condition was applied at *r* = 0:

$$u_{r}=0,\;\partial /\partial r=0,\;\;\text{where}\;\;\;=\;u_{r},u_{z},p,k,\omega$$

The interfaces among gas, metal and slag are assumed to be fixed in space. The free surface displacement is disregarded, since the stratification of the phases in the Earth’s gravitational field is assumed to prevent significant interfaces displacement. Slip boundary condition is imposed on the upper metal surface justified by the fact that air above the metal has significantly lower viscosity and exerts negligible stress on the metal surface:

$$\text{u}\cdot \text{n}=0,\;{\text{K}}_{\text{n}}-({\text{K}}_{\text{n}}\cdot \text{n})\text{n}=0,\;{\text{K}}_{\text{n}}=\text{K}.\text{n}$$

∇k⋅n=0,∇ω⋅n=0

A no-slip condition is imposed on the metal-slag (MS) and metal–refractory (MR) interfaces to ensure tangential momentum transfer between the phases. The metal moves tangentially to the interface with the velocity of slag **u**_*s*_ at the MS interface or with the velocity of the rotating refractory **u**_*rot*_ at the MR interface owing to TBRC rotation about its axis of symmetry:

$$\text{u}\cdot \text{n}=0,\;\text{K}\cdot \text{n}=-\rho u*u_{\tau }\;{\text{u}}_{\tau }/|{\text{u}}_{\tau }|,\;\nabla k\cdot \text{n}=0$$

$$\omega =(\omega_{visc}^{2}+\omega_{\text{log}}^{2}{)}^{1/2},\;\omega_{visc}=6\mu /(\rho \beta_{0}\delta_{w}^{2}),\;\omega_{\log}=u*/(\kappa_{\text{v}}\delta_{w}\sqrt{\beta_{0}^{*}})$$

$${\text{u}}_{\tau }={\text{n}}_{rel}-({\text{u}}_{rel}\cdot \text{n})\text{n},\;{\text{u}}_{rel}=\text{u}-{\text{u}}_{bnd},\;{\text{u}}_{bnd}=\{\begin{array}{l} {\text{u}}_{s}\;,\;\;\text{at}\;\text{MS}\;\text{interface}\\ {\text{u}}_{rot}\text{,}\;\;\text{at}\;\text{MR}\;\text{interface} \end{array}$$

$$u_{rot,r}=0,\;\;u_{rot,\varphi }=r\omega_{rot},\;u_{rot,z}=0,\;\;u*=[u_{\tau ,visc}^{4}+(u_{\text{log}}^{*}{)}^{4}{]}^{\frac{1}{4}},\;u_{\tau }=[u_{\tau ,visc}^{4}+u_{\tau ,\text{log}}^{4}{]}^{\frac{1}{4}}$$

$$u_{log}^{*}=\sqrt{\max(0,k)\sqrt{\beta_{0}^{*}}},\;u_{\tau ,visc}=\sqrt{\mu |{\text{u}}_{\tau }|/(\rho \delta_{w})},\;u_{\tau ,log}=|{\text{u}}_{\tau }|\kappa_{\text{v}}/(\text{ln}[\max(1,\delta_{w}^{+})]+B\kappa_{\text{v}})$$

$$\delta_{w}^{+}=\rho u*\delta_{w}/\mu ,\;\kappa_{\text{v}}=0.41,\;\text{B}=5.2$$

where **n** is the unit normal vector at the interface, *ω*_*rot*_ is the angular velocity of the TBRC rotation, and *δ _w_* is the wall lift-off distance, which equals half of the size of the near-wall element. Ambient pressure *p _amb_* was imposed at the right top corner of the metal domain.

The above no-slip boundary condition at the metal-slag interface together with the condition of tangential stress continuity (see the next subsection) result in a bi-directional coupling of fluid flow equations of slag and metal at the interface. The temperature dependence of material properties, such as density and viscosity, as well as heat advection and turbulent thermal conductivity result in a bi-directional coupling of flow and heat transfer equations.

### 4.2. Fluid dynamics in slag phase

Similar unsteady incompressible RANS equations with *k–ω* turbulence model were solved in the slag domain. The only difference is that in the solid or porous phase of the slag, the Darcy force **f**_*D*_ is added as follows:

$${\text{f}}_{D}=-\mu (\text{u}-{\text{u}}_{rot})/K_{perm}$$

where *μ* is the intrinsic slag viscosity, and *K _perm_* is the slag permeability (Kozeny-Carman model).

$$K_{perm}=K_{0}g_{l}^{3}/{\left(1-g_{l}\right)}^{2}$$

where *K*_0_ = 7 × 10 ^–9^ m ^2^ and *g _l_* is the liquid fraction. Additional source terms related to the Darcy force were also added to the right-hand side of the turbulence equations ^12^: –2 *μk*/ *K _perm_* in the *k* equation and –2 *μω*/ *K _perm_* in the *ω* equation. The boundary condition of tangential stress continuity and zero normal velocity was applied at the slag-metal interface as follows:

$${\text{σ}}_{\text{τ},slag}={\text{σ}}_{\text{τ},metal},\;{\text{σ}}_{\text{τ}}=\text{σ}-(\text{σ}\cdot \text{n})\text{n}$$

σ=[−pI+K]⋅n(samenforboththeslagandmetal)

u⋅n=0,∇k⋅n=0,∇ω⋅n=0

The use of this boundary condition is justified by the fact that slag moves owing to the shear stress of the tangential metal flow above it, while the interface displacement in the normal direction is neglected. Together with the no-slip boundary condition provided in the previous subsection, the above boundary conditions establish a closed and solvable system of equations at the metal-slag interface, also resulting in a bi-directional coupling of flow equations in slag and metal. The no-slip condition at the slag-refractory interface and the axial symmetry condition at *r* = 0 are imposed with equations similar to those in the previous subsection. The hydrostatic pressure of the metal layer was applied at the top-right corner of the slag domain. As stated in the previous subsection, the flow and heat transfer equations are bi-directionally coupled due to temperature-dependent properties of materials, such as density and viscosity.

### 4.3. Heat transfer with surface-to-surface radiation

We use the Apparent Heat Capacity method to model slag solidification. According to the method, the enthalpy of fusion is smoothly redistributed in a particular range of temperatures around the melting point and is included in the specific heat capacity at constant pressure *c _p_*, resulting in a modified heat capacity $c_{p}^{\prime }$ . In other materials, where phase change does not occur, the modified heat capacity $c_{p}^{\prime }$ coincides with *c _p_*. Thus, the following heat equation is solved in all domains:

$$\rho c_{p}^{\prime }(\partial T/\partial t+\text{u}\cdot \nabla T)+\nabla \cdot \text{q}=0$$

$$\text{q}=-(k+k_{T})\nabla T$$

where *T* is the temperature, *ρ* is the density, **u** is the velocity, **q** is the conductive heat flux, *k* and *k _T_* are the molecular and turbulent thermal conductivity, respectively.

$$k_{T}=c_{p}\mu_{T}/{\mathrm{Pr}}_{T}$$

where *μ _T_* is the turbulent viscosity and Pr _*T*_ is the turbulent Prandtl number, which is often modelled as a function of the molecular Prandtl number Pr ^13^. For simplicity, a constant value of the turbulent Prandtl number was adopted in this numerical study:

$${\mathrm{Pr}}_{T}=0.85$$

The heat transfer is strongly coupled with the fluid flow. Convective heat transfer is driven by the fluid motion, with velocity fields influencing temperature distribution. Additionally, the turbulence model accounts for enhanced thermal transport by incorporating turbulent thermal conductivity, which plays a key role in energy exchange within the system. On the other hand, the temperature dependence of material density and viscosity implies the influence of heat transfer on the fluid flow.

In the solid domains, the convective term ( **u** · ∇ *T*) and turbulent conductivity *k _T_* are omitted. In the solid phase of the slag layer, these terms are suppressed with the help of the Darcy force (see Subsection 4.2). The condition of axial symmetry at *r* = 0 is

∂T/∂r=0

Temperature is continuous on all material interfaces:

$$T_{up}=T_{down}$$

where subscripts *up* and *down* denote the upward and downward sides of the interface, respectively, *up* corresponds to the material to which the unit normal vector **n** is pointing. The condition of the heat flux continuity/discontinuity at the material interfaces is

$${\text{q}}_{up}\cdot \text{n}-{\text{q}}_{down}\cdot \text{n}=q$$

where *q* denotes the surface density of the interfacial heat source. In contrast to reference 23, where gas temperature is used as a boundary condition, in the present work the useful burner power is imposed on internal boundaries of the rotary converter. This helps to avoid artificial energy production on internal surfaces of the converter and ensures global energy conservation. Thus, the boundary heat source provided by an oxy-fuel burner on the internal surfaces of the TBRC is

$$-\text{q}\cdot \text{n}={\text{q}}_{burner}$$

where the unit normal vector **n** at the metal-air or refractory-air interface points into the air. Interfacial heat source due to the exothermic aluminothermic reaction at the metal-slag interface

$$-[{\text{q}}_{slag}-{\text{q}}_{metal}]\cdot {\text{n}}_{slag}=q_{reaction}$$

where **n**_*slag*_ is pointing to the metal and *q _reaction_* is the surface density of the reaction power computed from the reaction enthalpy and reaction time. The heat flux due to external natural convection is applied to the external TBRC boundaries as follows:

$$-\text{q}\cdot \text{n}=h(T_{amb}-T)$$

where **n** is the outward unit normal vector and *h* is the heat transfer coefficient, computed according to the empirical models of external natural convection. On the interfaces and boundaries that participate in the surface-to-surface radiation (all external and internal surfaces of TBRC, as well as interfaces of air gaps), an additional boundary heat source *q _r_* is imposed owing to thermal radiation absorption:

$$q_{r}=\varepsilon (G-\sigma_{SB}T^{4})$$

where *ε* is the hemispherical emissivity of the radiating surface, *σ _SB_* is the Stefan-Boltzmann constant, and *G* is the surface irradiance.

## 5. Material properties and parameters

### 5.1. Refractory materials and air

The thermal properties of all refractory materials were provided by MINTEK ^14^: VR 90B alumina-silicate was used for the refractory bricks (see Table 1), L-Cast 18 alumina-silicate for the castable refractory (see Table 2), and 1260 ST-RB ceramic fiber blankets for the insulation fiber (see Table 3).

**Table 1.** Properties of VR 90B refractory bricks.

Parameter	Value
Thermal conductivity at 1000°C	1.4 W/m/K
Bulk density	2900 kg/m ^3^
Mass fraction of: SiO _2_/ Al _2_O _3_/ Fe _2_O _3_/ TiO _2_/ CaO/ MgO/ Na _2_O/ K _2_O	9/ 90/ 0.2/ 0.2/ 0.1/ 0.1/ 0.2/ 0.2 wt%

**Table 2.** Properties of L-Cast 18 castable refractory.

Parameter	Value
Thermal conductivity at 1000°C	2.8 W/m/K
Bulk density	2940 kg/m ^3^
Mass fraction of: SiO _2_/ Al _2_O _3_/ Fe _2_O _3_/ TiO _2_/ CaO/ MgO	5.1/ 92.2/ 0.1/ 0.8/ 0.9/ 0.9 wt%

**Table 3.** Properties of 1260 ST-RB ceramic fibre blanket.

Parameter	Value
Thermal conductivity at: 600/ 800/ 1000°C	0.15/ 0.22/ 0.30 W/m/K
Bulk density	128 kg/m ^3^
Mass fraction of SiO _2_/ Al _2_O _3_	57/ 43 wt%

The specific heat capacity at constant pressure *c _p_* ( *T*) is approximated based on materials composition:

$$c_{p}(T)=\sum_{i}\omega_{i}c_{p,i}(T),\;i={\text{SiO}}_{2},{\text{Al}}_{2}{\text{O}}_{3},\text{etc}.$$

where *ω _i_* is the mass fraction and *c _p,i_* ( *T*) is the specific heat capacity of component *i* from the NIST database ^15^. The thermal conductivity *k*( *T*) of the 1260 ST-RB ceramic fiber blanket was modelled as a function of temperature by linear interpolation between the given data points. A constant-value extrapolation of all properties was used for out-of-range temperatures. The air gaps (see Figure 1) are assumed to be filled with air, and their properties are taken from the COMSOL Multiphysics ^®^ materials library.

### 5.2. Slag and metal

In the metal phase, initially pure Al becomes an Al-Si-Ca alloy as it reacts with the slag. Similarly, initially SiO _2_-CaO slag becomes Al _2_O _3_-SiO _2_-CaO slag as it reacts with the metal. Thus, the slag and metal densities are modelled as functions of the temperature *T* and composition *X _i_* (mole fraction of component *i*):

$$\rho (T,X_{i})=\sum_{i}\left[X_{i}M_{i}\right]/\left(\sum_{i}\left[X_{i}V_{mol,i}(T)\right]+V^{EX}\right)$$

where *M _i_* and *V _mol,i_* are the molar mass and molar volume ^16– 19^ of component *i*, *V ^EX^* is a corrective interaction term ^19^, *i* = Al, Si, Ca for metal, and *i* = Al _2_O _3_, SiO _2_, CaO for slag. The composition of the slag and metal is modelled as a function of the relative reaction extent *ξ _rel_* which equals 0 at the beginning of the process and 1 at the end of the reaction:

$$X_{i}=X_{i,init}+\xi_{rel}\left(X_{i,final}-X_{i,init}\right)$$

For simplicity, *ξ _rel_* is a linear function of time:

$$\xi_{rel}=t/t_{r}$$

where *t _r_* is the user-defined reaction time. The initial *X _i,init_* and final *X _i,final_* compositions, as well as the reaction energy, were estimated using the software provided by the project partner SINTEF. This artificial neural network-based tool ^20, 21^ was trained on FactSage ^®^ data for the metal-slag system of interest. Other metal properties, such as thermal and electrical conductivity ^16^, dynamic viscosity ^22^, and heat capacity at constant pressure, were computed as for pure aluminum. The thermal conductivity of the slag was assumed to be constant and equal to 1 W/m/K . The slag viscosity was modelled as a function of the temperature and slag composition according to the mathematical model ^21^ of Kai Tang from SINTEF. Other slag properties, such as heat capacity, enthalpy, and electrical conductivity, were computed as functions of temperature and composition, according to the Ken Mills model ^23^.

## 6. Numerical results and discussion

### 6.1. TBRC preheating

**Figure 4.**
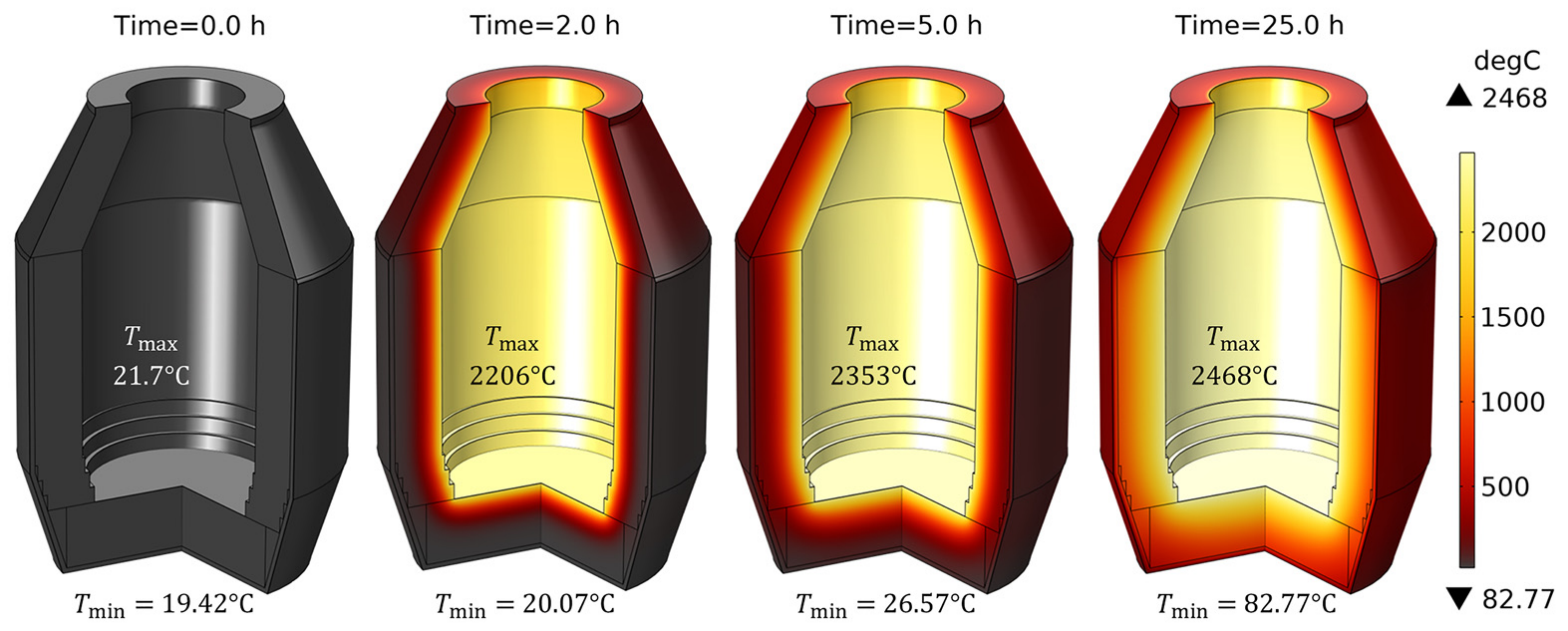
Time evolution of the temperature field at 600 kW burner power in an empty TBRC.

**Figure 5.**
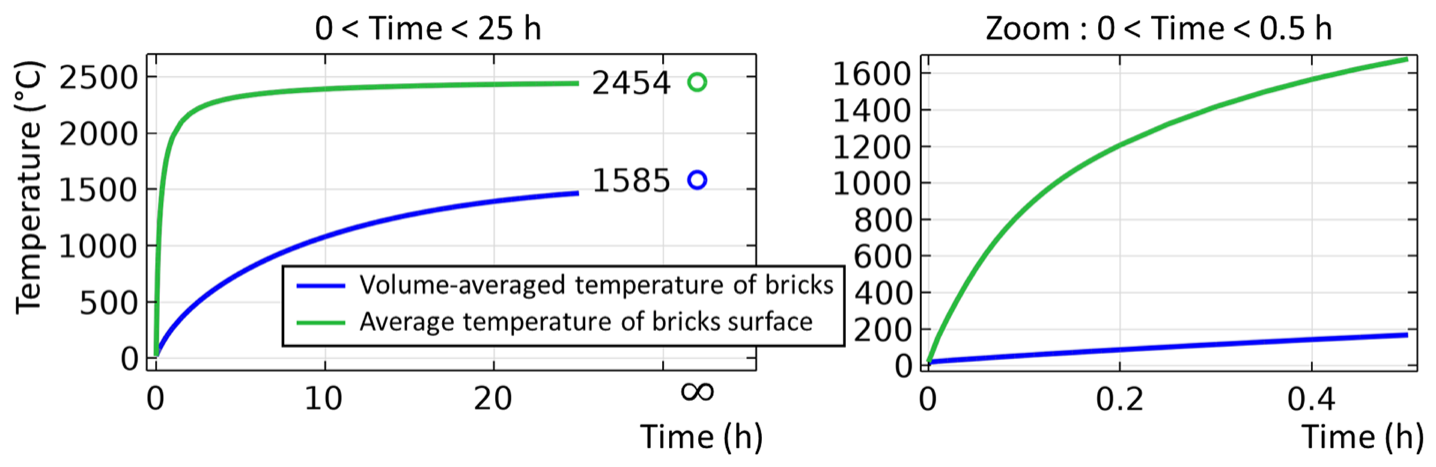
Average temperature of refractory bricks during 25 hours of preheating at 600 kW burner power.

**Figure 6.**
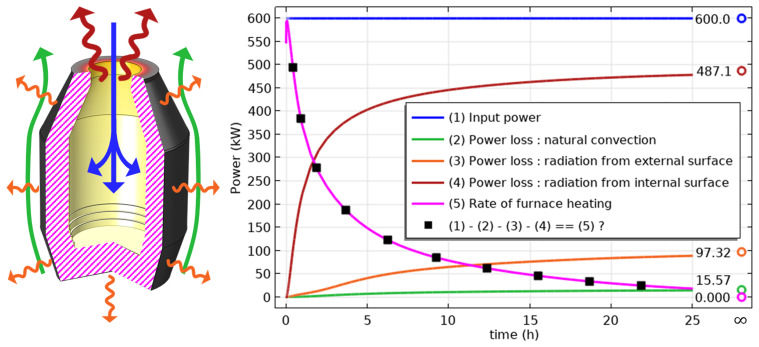
Left: heat fluxes contributing to the power balance. Right: time evolution of the power balance.

**Figure 7.**
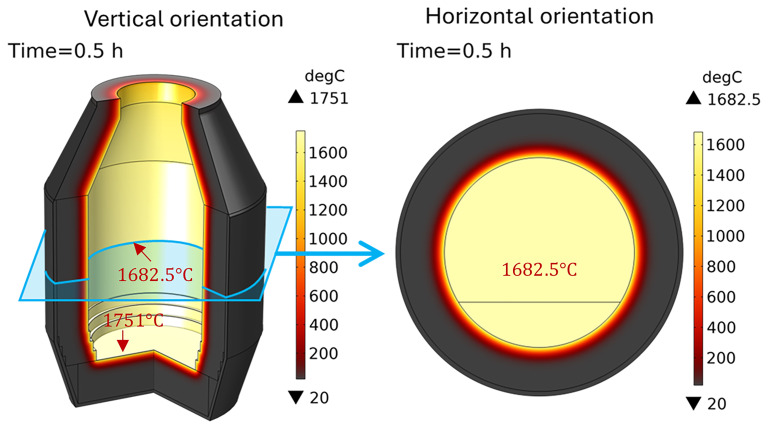
TBRC after 30 min of preheating with 600 kW burner power, initial condition for further computations.

The unsteady process of empty TBRC preheating with an oxy-fuel burner was numerically modelled, as shown in Figure 4. It was found that the surface of the refractory bricks (inside the TBRC) could reach the desired temperature of 1650°C in less than 30 min when the useful burner power was 600 kW, as shown in Figure 5. At the same time, the volume-averaged temperature of the bricks at *t* = 0.5 h stays below 200°C, meaning that the refractory volume remains relatively cold with respect to the internal TBRC surface. Further preheating resulted in a significant temperature increase, as shown in Figure 5, where the average temperature of the brick surface reached 2400°C after 12 h of preheating and 2454°C in the steady state (as *t* → ∞). Fig. Figure 6 shows the power balance of the system. The only heat source in the furnace was the oxy-fuel burner with a constant input power of 600 kW (blue line and arrows in Figure 6). Power is lost by three principal mechanisms: external natural convection (green line and arrows in Figure 6), thermal radiation from the external surface of the TBRC (orange line and arrows in Figure 6), and thermal radiation from the inside of the TBRC through the opening at the top of the furnace (red line and arrows in Figure 6). Black square markers represent the difference between the input power and sum of the power losses. As can be seen in Figure 6 (right), a perfect power balance is numerically achieved for all simulated times: black square markers coincide with the rate of furnace heating (see magenta line in Figure 6), which is computed as follows:

$$\text{rate}\;\text{of}\;\text{furnace}\;\text{heating}=\int_{V}\rho c_{p}^{\prime }(T)\frac{\partial T}{\partial t}dV$$

where integration is performed over the entire TBRC volume, *V*. At the beginning of preheating ( *t* = 0), all types of power losses were small because the TBRC was still cold. Thus, at *t* = 0 the entire burner power (600 kW) was spent on furnace heating (magenta line in Figure 6). As the furnace body became hotter with time, the power losses increased. It was found that at steady state ( *t* → ∞), most of the power (81.2%) was lost in the form of thermal radiation from the internal surface of the TBRC towards the ambient environment through the opening at the top of the furnace. Therefore, the use of a furnace lid is recommended to prevent significant heat loss during operations that do not involve charging, tapping, or heating with a burner. The TBRC state after 30 min of preheating with 600 kW input power (see Figure 7) served as the initial condition for further computations with a furnace charge.

### 6.2. Influence of TBRC rotation frequency

**Figure 8.**
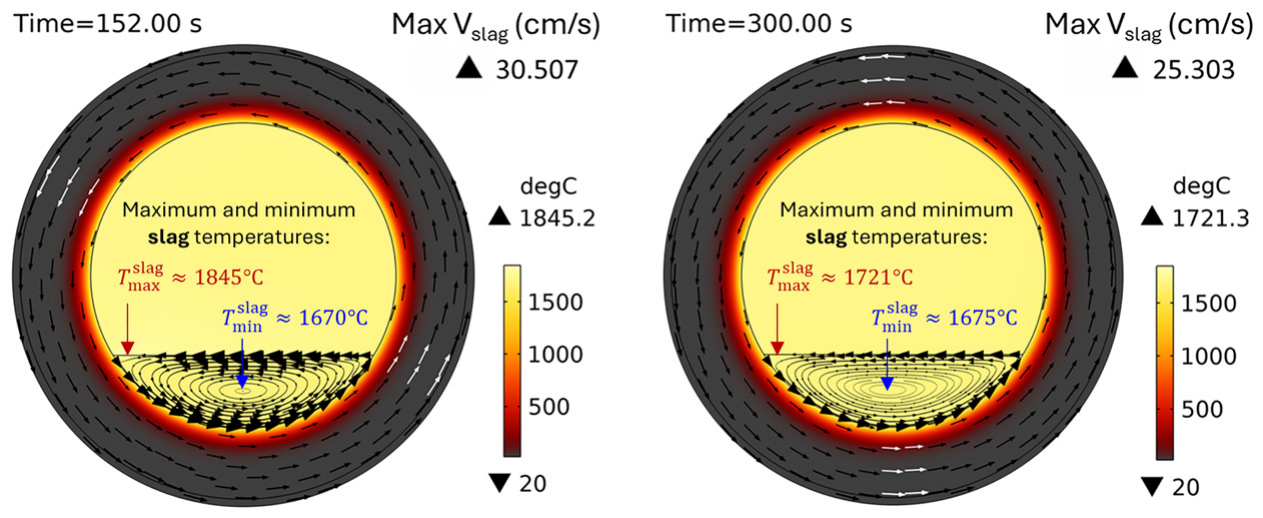
Temperature field and slag velocity at 5 rpm and 600 kW burner power in a horizontal TBRC.

**Figure 9.**
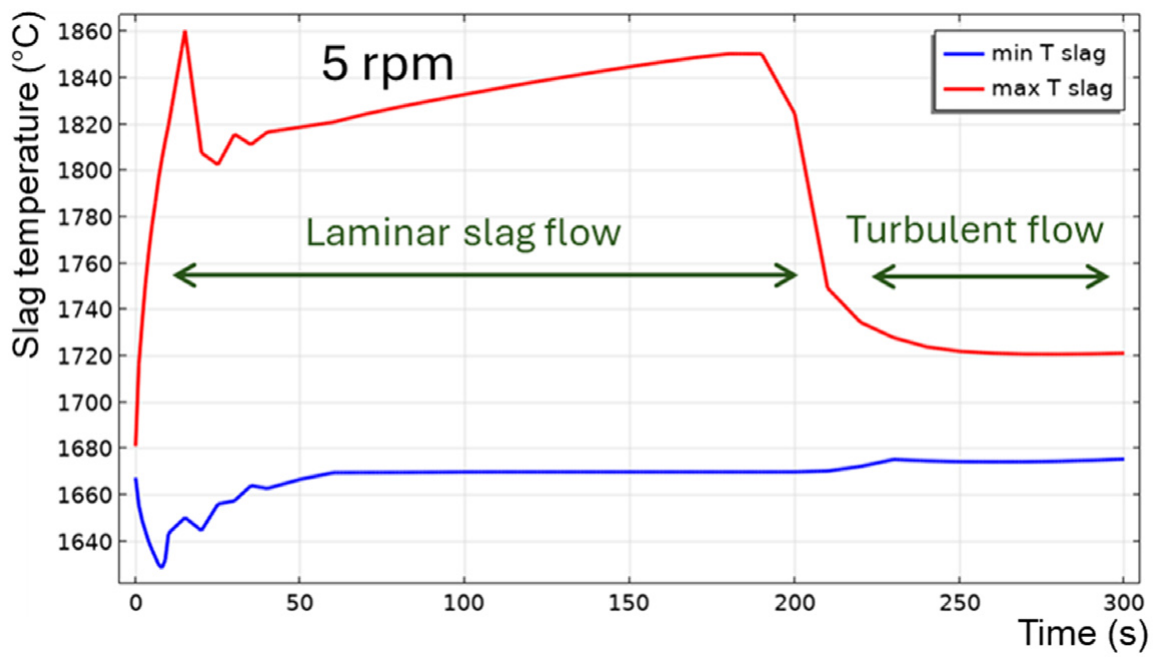
Maximum and minimum slag temperature at 5 rpm and 600 kW burner power in a horizontal TBRC.

Let us consider TBRC charged with only slag that rotates in its horizontal position ( *θ* = 0, see Figure 2). This configuration of the furnace was simulated using a 2D plane model that represents the TBRC cross-section, as shown in Figure 2 (b). The initial slag temperature was 1670°C. The initial temperature field in the TBRC refractory and air above the slag were taken from the previously solved preheating problem, as shown in Figure 7 (right). The dominant heat transfer mechanism in the air phase was surface-to-surface radiation. Therefore, to simplify the problem, air convection was not solved, and only air conduction was considered. The TBRC rotation frequency is initially zero (0 rpm at *t* = 0) and then smoothly increases to a given value during the first 10 s of the simulated process. The temperature field and slag velocity for a full burner power (600 kW) and a rotation frequency of 5 rpm are shown in Figure 8. The maximum temperature is located on the slag surface exposed to burner heating, while its minimum is always on the external surface of the TBRC refractory. The corresponding time evolution of slag temperature is shown in Figure 9. The minimum and maximum temperatures are indicated by the blue and red lines, respectively.

**Figure 10.**
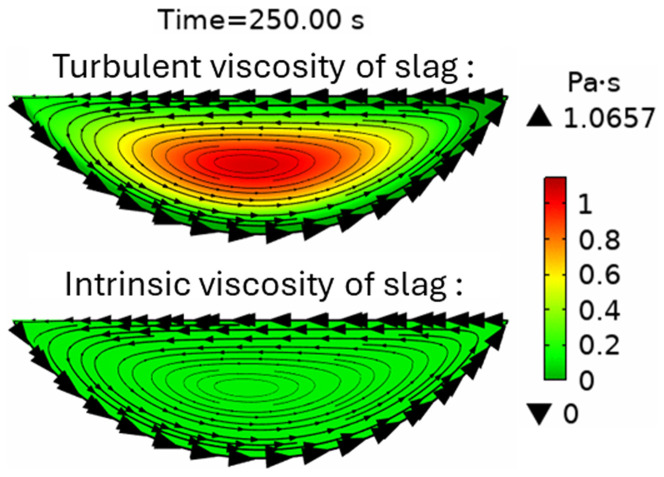
Intrinsic and turbulent slag viscosity at t = 250 s at 5 rpm and 600 kW burner power in a horizontal TBRC.

For *t* < 20 s, the flow is not yet fully established in the entire slag volume owing to the recent activation of TBRC rotation. This resulted in poor slag stirring during the first 20 s and, consequently, poor thermal conductivity of the slag layer. Thus, its maximum temperature *T _max_* quickly rises to 1860°C on the surface heated by the burner, and its minimum temperature *T _min_* quickly reduces to 1630°C at the bottom of the slag layer owing to heat losses to the ambient environment through the refractory. At *t* = 20 s, when the slag flow was fully established in the entire slag volume, more intensive stirring resulted in a decrease of *T _max_* down to 1810°C and an increase of *T _min_* up to 1650°C. Between 50 and 200 s, the maximum slag temperature slowly increases on its surface because of burner heating, whereas its minimum temperature remains almost constant and equal to the initial slag temperature *T*_0, *s*_ = 1670°C. This minimum is found in the middle of the flow vortex, where the velocity is low and heat transfer occurs primarily through conduction. Thus, the low intrinsic thermal conductivity of the slag results in very slow temperature changes in the middle of the flow vortex. At 200 s, there was a qualitative change in the temperature behavior: the difference between *T _max_* and *T _min_* suddenly decreased. This can be explained by the spontaneous transition to a turbulent flow regime. Indeed, the turbulent slag viscosity *μ _T_* is zero for *t* < 200 s, whereas the intrinsic viscosity (≈0.123 Pa⋅s) dominates, which corresponds to a laminar flow regime. However, after 200 s, the turbulent viscosity (≈1 Pa⋅s) dominated the intrinsic viscosity (≈0.117 Pa⋅s), as shown in Figure 10, which corresponds to a turbulent flow regime.

**Figure 11.**
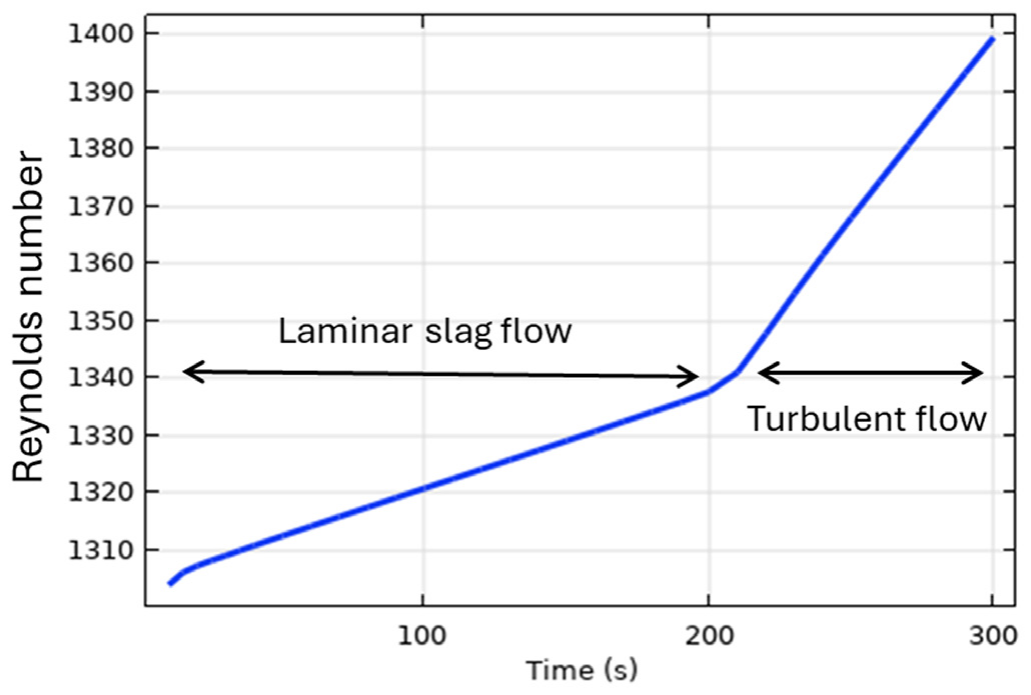
Reynolds number for slag at 5 rpm and 600 kW burner power in a horizontal TBRC.

In general, turbulence intensifies the heat transfer in the bulk of the fluid owing to turbulent mixing at multiple length scales. In the present numerical model, this effect causes a reduction in the temperature difference between *T _max_* and *T _min_* in the slag layer for *t* > 200 s. Qualitatively, the transition to a turbulent flow regime can be explained using the Reynolds number:

Re⁡=ρuL/μ=ρRintωrotHs/μ

where *ρ* is the slag density, *L* is the characteristic length of the problem (height of the slag layer in this case *L* = *H _s_*), *u* = *R _int_ω _rot_* is the flow velocity, *R _int_* is the internal radius of the TBRC, and *ω _rot_* = 2 *π* ⋅5 rpm = 0.52 rad/s is the angular velocity of the TBRC rotation. As the slag is continuously heated by the burner, its average temperature increases, resulting in a reduction in its average intrinsic viscosity *μ*. This leads to a continuous increase in the Reynolds number, as shown in Figure 11, until it reaches a threshold value (1340 in this case) that triggers turbulence. The exact threshold value can be a complicated function of problem parameters and geometry.

**Figure 12.**
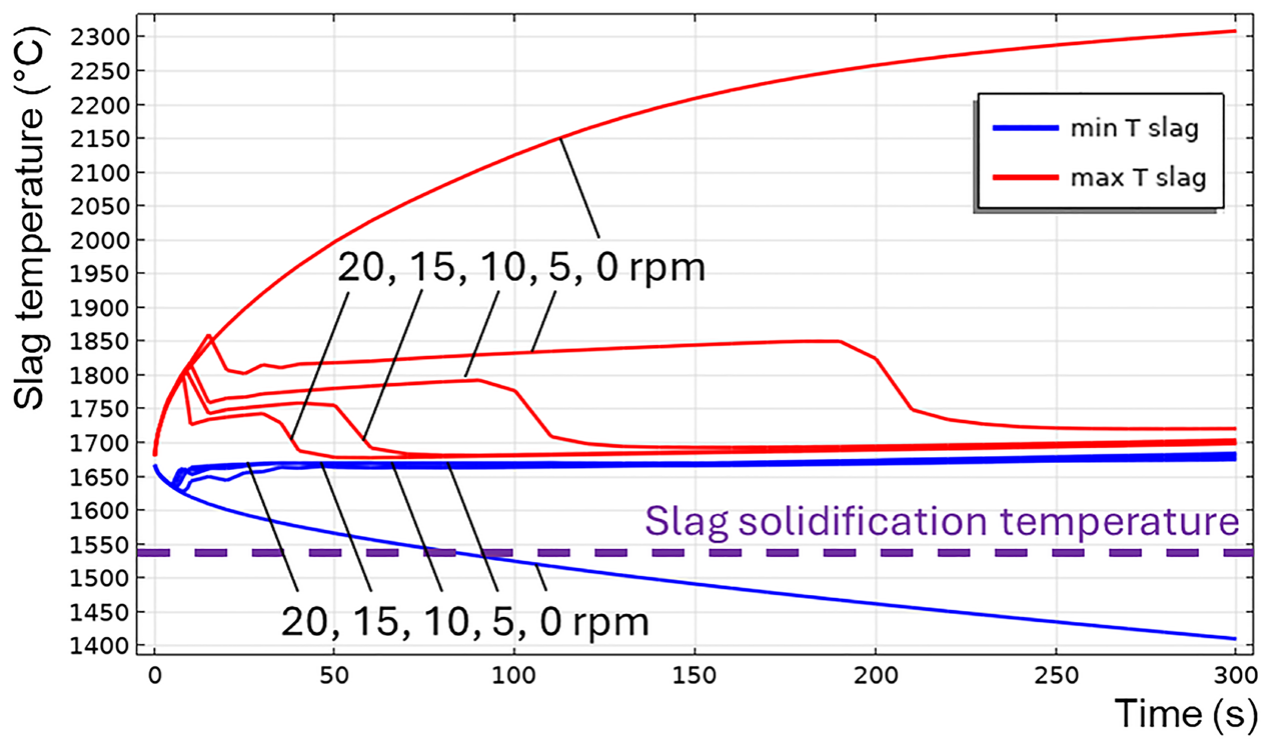
Maximum and minimum slag temperature at different rotation frequencies and 600 kW burner power in a horizontal TBRC.

**Figure 13.**
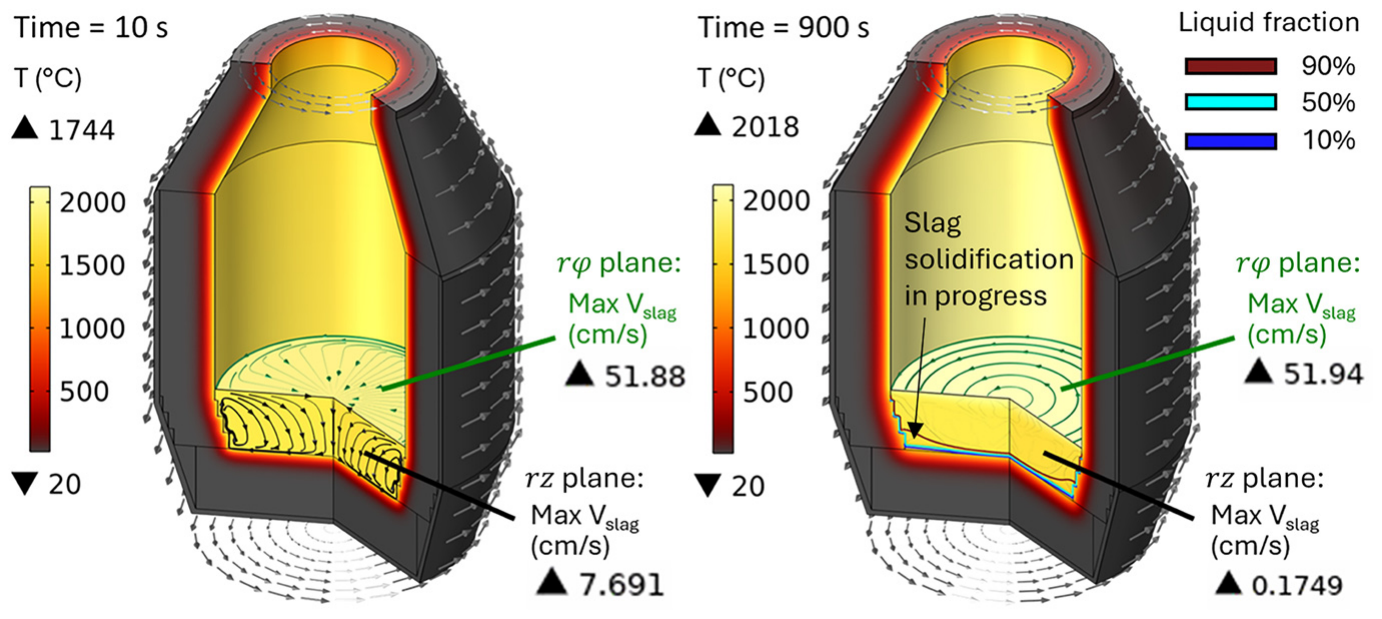
Temperature field and slag velocity at 10 rpm and 600 kW burner power in a vertical TBRC.

**Figure 14.**
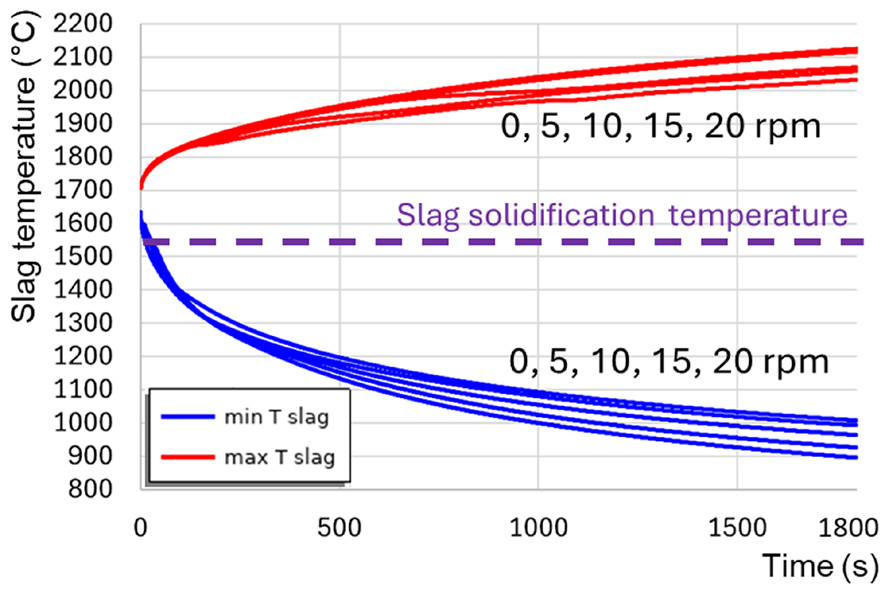
Maximum and minimum slag temperature at different rotation frequencies and 600 kW burner power in a vertical TBRC.

The transition to a turbulent flow regime occurs spontaneously, similar to an avalanche (see *T _max_* at *t* = 200 s in Figure 9), because of the positive feedback loop: the intensification of turbulence results in the intensification of heat transfer, which increases the average slag temperature and reduces its average intrinsic viscosity, which further intensifies the turbulence. When TBRC rotates faster, the Reynolds number increases; therefore, the spontaneous transition to a turbulent flow regime occurs earlier (see the 5–20 rpm cases in Figure 12). When the TBRC does not rotate, only natural convection due to buoyancy forces in Earth’s gravity is present in the slag layer. In this case, the slag velocity was much lower than that in the forced-convection case. Thus, poor slag mixing results in a significant temperature difference across the layer (see the 0 rpm case in Figure 12) and slag freezing at the bottom of the pool (see the dashed line in Figure 12 representing the slag solidification temperature). Let us now consider the influence of the rotation frequency in vertically oriented TBRC ( *θ* = 90°) (see Figure 2 (a)). The temperature and velocity fields are shown in Figure 13 for a rotation frequency of 10 rpm at *t* = 10 and 900 s (note that the total simulated duration was 1800 s). Slag velocity in the *rφ* plane reaches 52 cm/s due to furnace rotation, whereas in the *rz* plane it remains below 0.18 cm/s for *t* ≥ 900 s. This is explained by the fact that, in vertical TBRC, slag rotates together with TBRC about its axis of symmetry, whereas its vertical position is not perturbed by rotation. In contrast, in horizontally oriented TBRC, as shown in Figure 8, when the furnace rotates, it drags slag up against the gravity force, which in turn brings it down to the original level and therefore induces stirring. Thus, in vertical TBRC, any rotation frequency is insufficient for slag stirring, resulting in a significant temperature difference across the slag layer (see Figure 14). As a result, slag freezing occurs at the bottom of the pool (see the dashed line in Figure 14) representing the slag solidification temperature and liquid fraction isolines in Figure 13). The above results show that a vertical TBRC orientation is most unfavorable for slag stirring and leads to its solidification, whereas a horizontal TBRC orientation is the most favorable for stirring and works well as long as the furnace rotates, even at 1 rpm.

### 6.3. Influence of the burner power and of TBRC inclination angle

**Figure 15.**
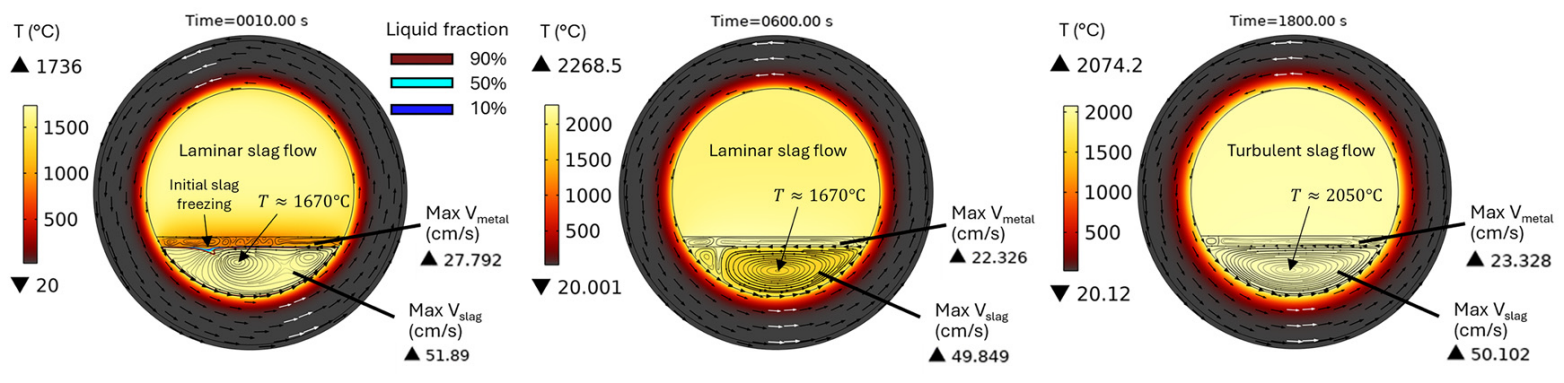
Temperature and velocity fields at 10 rpm and 600 kW burner power in a horizontal TBRC.

**Figure 16.**
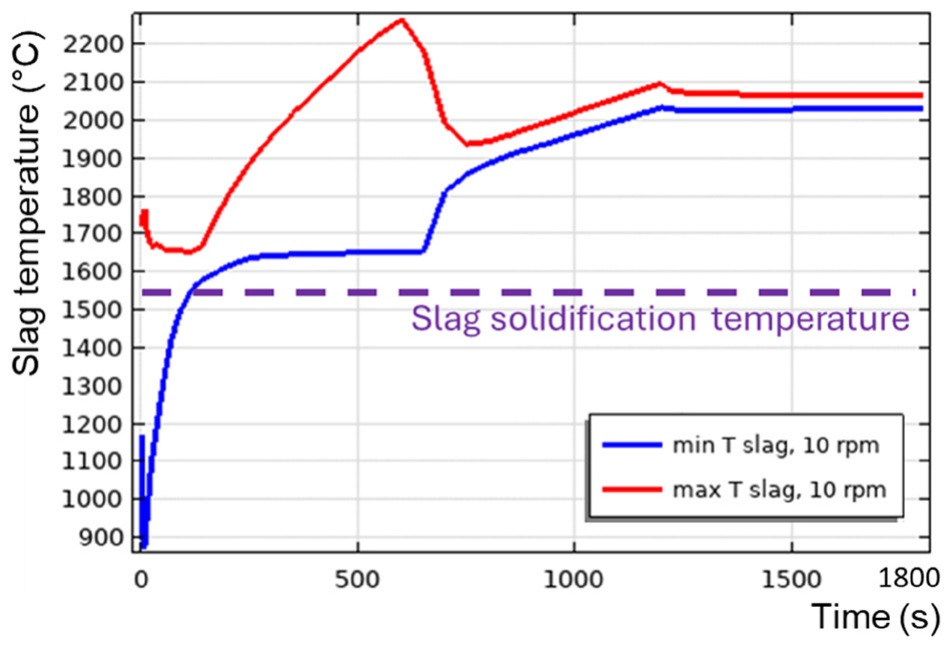
Maximum and minimum slag temperature at 10 rpm and 600 kW burner power in a horizontal TBRC.

In this subsection, a TBRC charged with slag and metal is numerically modelled, considering an additional heat source at the metal-slag interface owing to aluminothermic reduction. Experimental investigations ^24^, carried out in the framework of the SisAl Pilot project, have indicated that the oxygen flux across the metal-slag interface, associated with the aluminothermic reaction, drops significantly after first 20 minutes of the reduction process. More specifically ^24^, the average flux was estimated to be 10.3–15.4 g⋅m ^–2^⋅s ^–1^ within 3 min of the holding time, 3.6 g⋅m ^–2^⋅s ^–1^ between 3–20 min and 0.1 between 20–40 min at 1650°C. Thus, the first 30 min of the process were modelled, keeping in mind that aluminothermic reduction was assumed to last for 20 min, followed by 10 min of additional time needed for the TBRC tapping. The objective was to determine the occurrence of slag solidification depending on the burner power and TBRC inclination angle. Regarding the last one, vertically oriented TBRC ( *θ* = 90°, see Figure 2) can be modelled with a 2D axisymmetric model, and horizontally oriented TBRC ( *θ* = 0°) with a 2D plane model, whereas any other angle (0° < *θ* < 90°) requires a full 3D model. To be efficient with respect to the computation time, 2D models are prioritized over 3D models. Therefore, interpolation of the major quantities of interest between *θ* = 0° and 90° is performed instead of 3D modelling. In addition, only one rotation frequency of 10 rpm, as recommended by MINTEK, was modelled here. The temperature and velocity fields in a horizontal TBRC are shown in Figure 15 for a 600 kW burner power. The corresponding time evolution of the maximum *T _max_* and minimum *T _min_* slag temperatures is shown in Figure 16. Initially, *T _min_* drops to 850°C due to direct contact with the metal, whose initial temperature is 800°C. This causes slag solidification directly under the metal layer (see the liquid fraction isolines in Figure 15 at *t* = 10 s). During the next 200 s, owing to the reaction heat, *T _min_* increased back to the initial slag temperature *T*_0, *s*_ = 1670°C (see Figure 16). At this point, the previously frozen slag is fully remelted without any risk to furnace operation. Further TBRC heating increases *T _max_* at the metal-slag interface to 2280°C. At the same time, *T _min_* in the middle of the larger laminar convective vortex (see *t* = 600 s in Figure 15) remains equal to *T*_0, *s*_ owing to the poor mixing. At *t* ≈ 700 s, see Figure 16, a transition to a turbulent flow regime occurs in the slag, resulting in a significant turbulent thermal conductivity that homogenizes the temperature across the slag layer and brings *T _min_* and *T _max_* closer. Further heating increased both *T _min_* and *T _max_* until the chemical reaction was complete at *t* = 1200 s. Subsequently, during 1200 s < *t* < 1800 s, the slag temperature was maintained at an almost constant value owing to the operating burner and turbulent mixing in the slag layer. Thus, there is no risk of slag solidification if the horizontally oriented TBRC operates at 10 rpm and 600 kW burner power.

**Figure 17.**
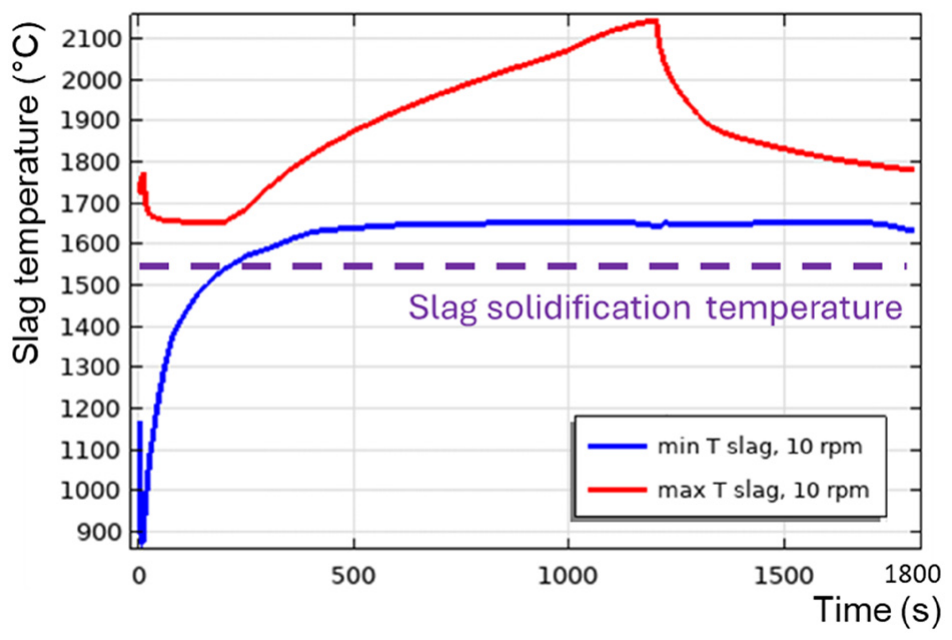
Maximum and minimum slag temperature at 10 rpm and 164 kW burner power in a horizontal TBRC.

Now, let us reduce the burner power. Figure 17 presents *T _min_* and *T _max_* in a horizontally oriented TBRC at 10 rpm and 164 kW burner power. This is the adjusted burner power that is sufficient to maintain the slag in a liquid state during the first 30 min of the process. A transition to a turbulent flow regime was not observed in this case. *T _max_* increased up to 2150°C owing to the reaction heat and then dropped at 1200 s as the reaction stopped. *T _min_* in the middle of the convective vortex remains equal to *T*_0, *s*_ until *t* = 1700 s, and then starts to decrease owing to heat losses but does not reach the solidification temperature *T _sol_* = 1540°C during the simulated times. Therefore, there was no risk of slag solidification in this furnace configuration.

**Figure 18.**
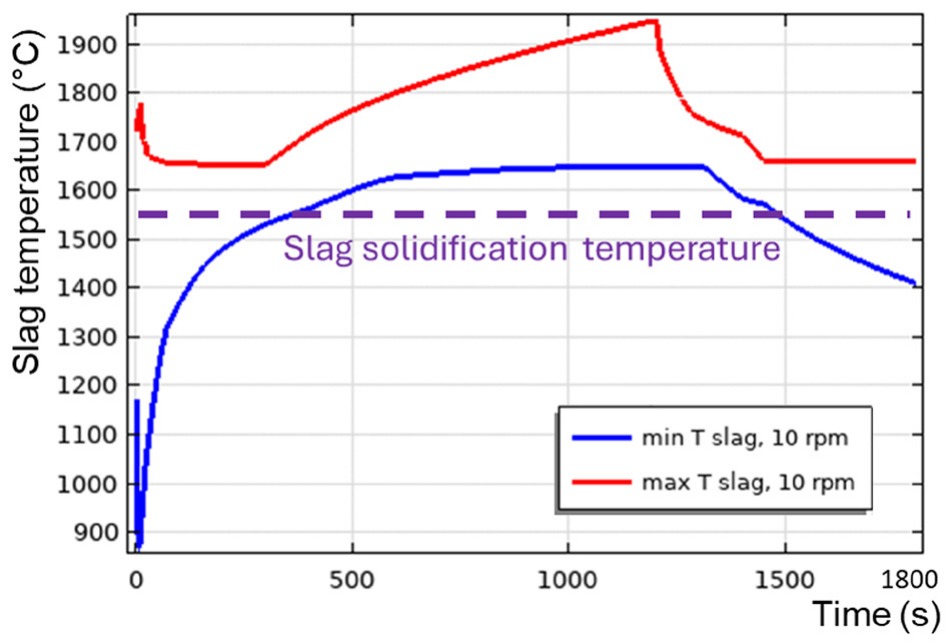
Maximum and minimum slag temperature at 10 rpm and 0 kW burner power in a horizontal TBRC.

Now, let us remove the burner and see if only the aluminothermic reduction power is sufficient to maintain the slag in a liquid state. The time evolution of slag temperature is shown in Figure 18. As one can see, the slag starts to solidify very quickly after the end of the reaction (see *t* = 1500 s in Figure 18). As before, the slag temperature in the middle of the convective vortex remained equal to the initial slag temperature *T*_0, *s*_. In Figure 18 the mid-vortex temperature is represented by *T _min_* for 500 s < *t* < 1300 s and by *T _max_* for *t* > 1500 s. At *t* = 1800 s, slag solidification occurs primarily on the slag-metal and slag-refractory interfaces, resulting in the formation of a freeze-lining. Thus, there was a risk of slag solidification in this case.

**Figure 19.**
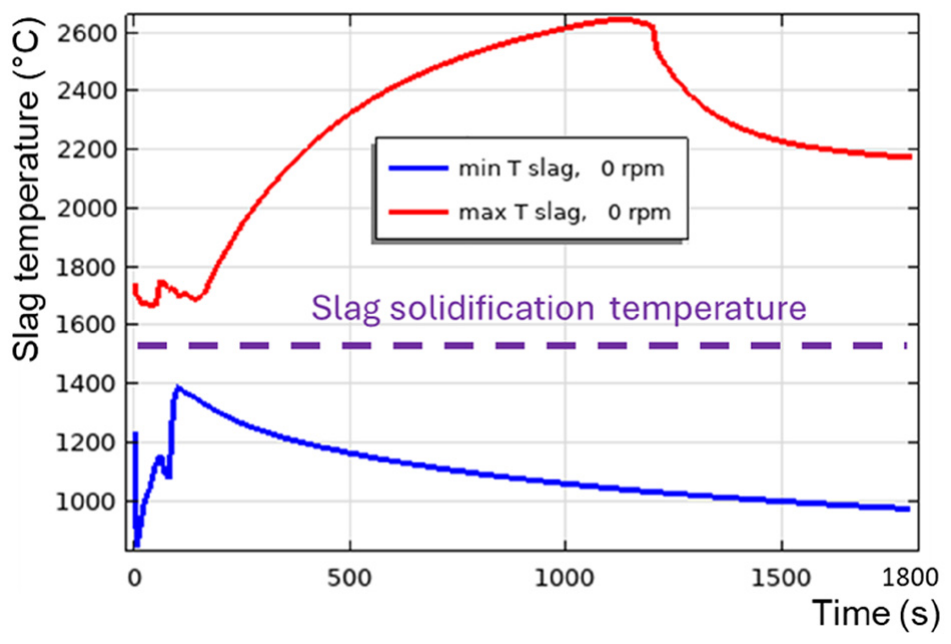
Maximum and minimum slag temperature at 0 rpm and 600 kW burner power in a vertical TBRC.

**Figure 20.**
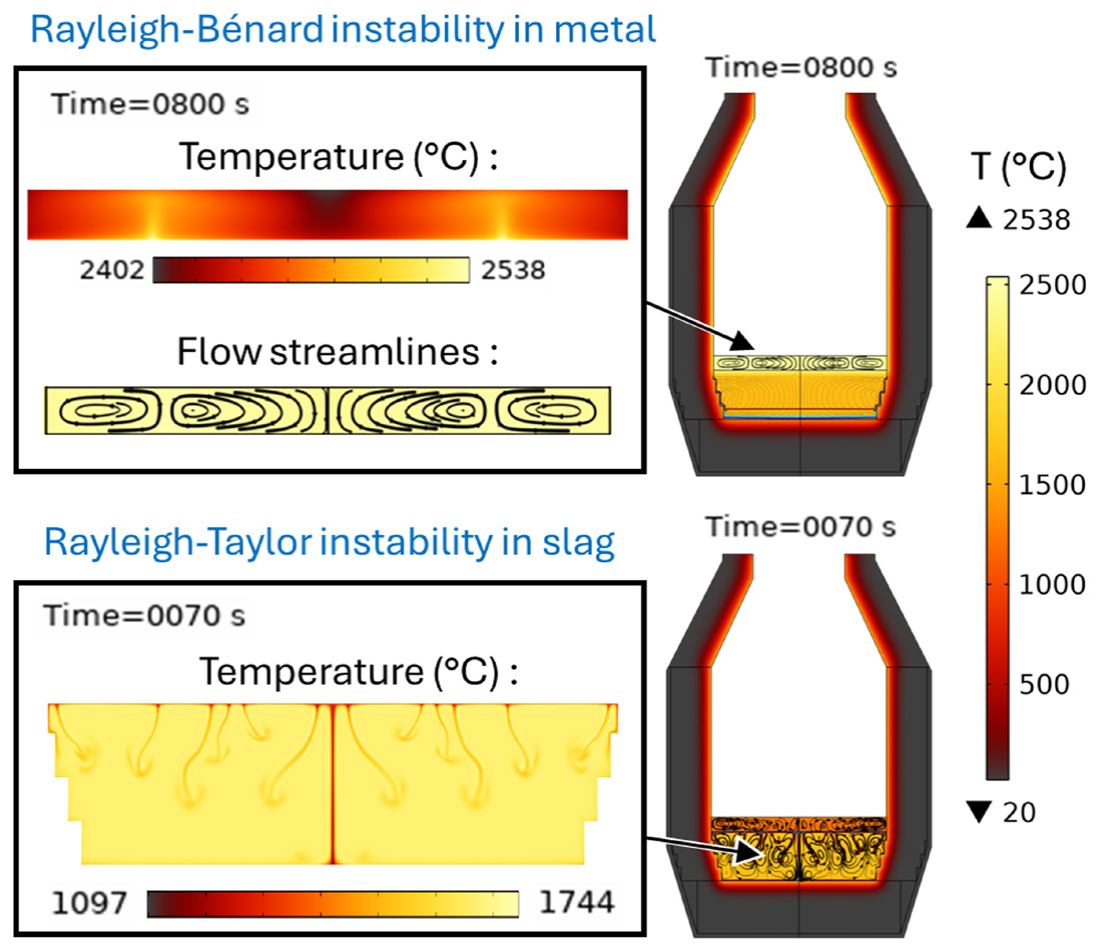
Rayleigh-Bénard instability in the metal phase and Rayleigh-Taylor instability in the slag phase at 0 rpm and 600 kW burner power in a vertical TBRC.

Regarding the vertical TBRC orientation, as shown above, even with a maximum burner power of 600 kW, the slag solidifies at the bottom of the pool owing to inefficient stirring. Even the presence of additional heating at the metal-slag interface due to aluminothermic reduction is not sufficient to keep the slag in a liquid state, as shown in Figure 19. Note that the 0 rpm case is modelled here, as *T _min_* and *T _max_* are essentially independent of the rotation frequency in the vertical TBRC, as shown in Figure 14. The existence of the Rayleigh-Bénard instability in the metal layer and of the Rayleigh-Taylor instability in the slag is demonstrated in Figure 20.

**Figure 21.**
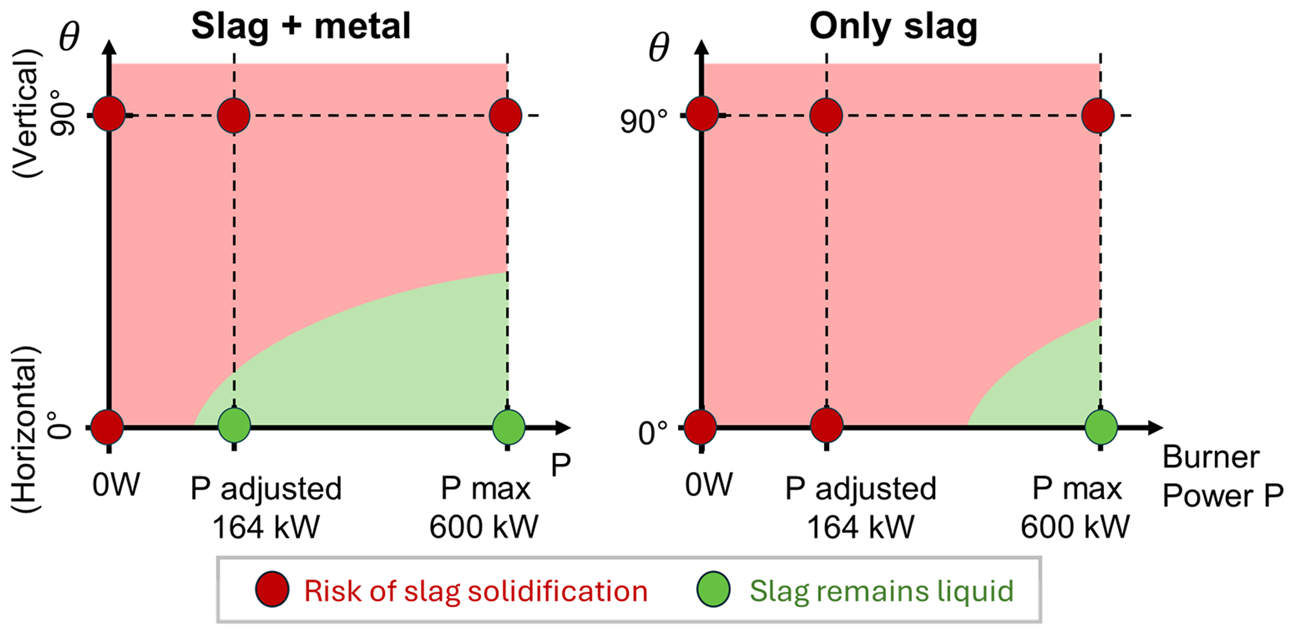
Occurrence of slag solidification depending on the burner power and on the TBRC inclination angle.

All the above results are summarized in a 2D diagram of the occurrence of slag solidification depending on the burner power and TBRC inclination angle, as shown in Figure 21 (left). In this diagram, the location of the border between the red zone (“Risk of slag solidification”) and green zone (“Slag remains liquid”) is defined by condition *T _min_* = *T _sol_*, where *T _sol_* = 1540°C is the slag solidification temperature, and *T _min_* is the minimum slag temperature at *t* = 1800 s obtained by interpolation between available diagram points. Finally, if the metal is not charged into the TBRC and only slag is present, the absence of heat from the aluminothermic reduction is compensated by the increase in the burner power, which shifts the above diagram to the right, as shown in Figure 21 (right).

The above-presented modelling approach and numerical findings suggest the following general takeaways that can inform decision-making and process improvements in similar TBRC operations:

- Reducing the TBRC inclination angle *θ*, so that its axis of rotation is getting closer to a horizontal orientation, improves the charge mixing and reduces the risk of slag solidification.
- In the limiting case of a horizontally oriented TBRC ( *θ* = 0), slag solidification can be effectively avoided even at a rotation frequency as low as 1 rpm.
- The optimum burner power, specific to each TBRC design and refractory, can be determined through numerical modelling to prevent charge solidification.
- Once the optimum burner power is identified for a given charge composition, it can be adjusted for other compositions by accounting for the slag-metal reaction power, estimated from reaction energy and empirical reaction time.
- By increasing the charge temperature and the TBRC rotation frequency, a particular threshold Reynolds number can be reached, above which the flow transitions to a turbulent regime, which significantly improves the charge mixing and increases the rate of chemical reaction.

## 7. Conclusions

Numerical modelling of the top-blown rotary converter (TBRC) designed by MINTEK was presented. It is shown that the internal surface of TBRC can be preheated to 1650°C in less than 30 min if the useful burner power is 600 kW. Most of the thermal energy (81.2% in the steady state) is lost by thermal radiation from the inside of the TBRC towards the ambient environment through the opening at the top of the TBRC. Thus, the use of a furnace lid is strongly recommended to prevent significant heat loss. The occurrence of slag solidification depending on the burner power and TBRC orientation was assessed in 2D diagrams. The model of the charged TBRC shows that the horizontal TBRC orientation is the most favorable for charge stirring. As long as it rotates even at 1 rpm, stirring is efficient for maintaining the slag in a liquid state. In contrast, in vertically oriented TBRC, stirring is not efficient at any rotation frequency (tested up to 20 rpm) and leads to slag solidification at the bottom of the pool. Even a maximum burner power of 600 kW, together with the aluminothermic reduction heat, is not sufficient to avoid slag solidification in a vertical TBRC. However, an optimum burner power of 164 kW exists in the horizontal TBRC. This power, together with the reaction heat, is sufficient to maintain the slag in a liquid state during the first 30 min of the process, if the furnace is horizontally oriented. The modelling work resulted in the creation of a powerful decision-making tool that plays a pivotal role in the comprehensive numerical support provided for the pilot campaign of the SisAl Pilot project. This tool permits exploration of new optimal configurations.

The broader-level findings can be summarized as follows. One can improve the charge mixing and reduce the risk of slag solidification by reducing the TBRC inclination angle *θ* and by imposing at least 1 rpm rotation frequency. The optimum burner power can be estimated based on a numerical simulation of a given TBRC design, combined with an empirical estimate of the chemical reaction power. At specific rotational speeds, a threshold Reynolds number can be exceeded, triggering a transition to turbulent flow that significantly enhances charge mixing and accelerates chemical reactions.

We acknowledge that experimental validation and comparison with established benchmarks would further strengthen our findings. We consider this to be an important direction for future work and encourage experimental studies to help validate our predictions.

## Data Availability

Repository: Zenodo. Dataset title: “Refractory materials used in the numerical model of TBRC”. https://doi.org/10.5281/zenodo.13880322 ^14^. This project contains the following underlying data: Data file 1: “Insulation fibre 1260 ST-RB.png” (Data sheet for the properties of 1260 ST-RB insulation fibre) Data file 2: “Refractory bricks VR 90B.png” (Data sheet for the properties of VR 90B refractory bricks) Data file 3: “Castable refractory L-Cast 18.png” (Data sheet for the properties of L-Cast 18 castable refractory) Data are available under the terms of the Creative Commons Attribution 4.0 International licence.
