# Supplementary material for: Numerical model of a top-blown rotary converter preheating and charge heating with an oxy-fuel burner
Source: Open Res Eur. 2025 Jul 4;4:248. Originally published 2024 Nov 8. [Version 2] doi: 10.12688/openreseurope.18594.2 (PMC12397741; doi:10.12688/openreseurope.18594.2)
Supplement: Peer review history

# Reviewer report: Reviewer response for version 2

**DOI:** 10.21956/openreseurope.22542.r57528

**Published:** 2025-09-02

**Authors:** Theresa Coetsee

**Affiliations:** 1 University of Pretoria, Pretoria, Gauteng, South Africa

The article describes the development of a CFD model of the heat transfer in a pilot plant rotary converter heated by a 600kW oxy-fuel burner. The main aim of the model is to serve as a plant design tool for construction and to direct process operational issues to be addressed during plant engineering design. In these aims, the model is successful.

 Literature: Add the following similar paper for completeness: [Reference 1]

 Sufficient details: Some slag-metal properties are derived from an artificial neural network-based tool based on FactSage data, which is not accessible to all. However, others can approximate these properties as well. 

 Technical questions: 

 1. The brick surface exceeds the refractory melting point in Figure 5, so the burner energy needs to be scaled back. Mention this. 

 2. According to section 4.3 , the method used to input the burner energy is simplified as a boundary condition. The CFD modelling of burner characteristics is quite complex and the flame profile and density influence the flame radiation efficiency. None of these effects are present in the current model. To better gauge the heat balance components in this model and future refinements, it is instructive to express heat transfer values in kW/m^2 over each surface, not only as kW as in Figure 6. A simple table with square meter areas and kW and resultant kW/m^2 for the steady-state conditions will be sufficient. In this way the design numbers can be compared to other models and the plant design can be easily scaled. 

 Although this is not a validated detailed model, it serves its purpose as stated in the introduction. Future validation against operational data will be interesting.

Is the study design appropriate and does the work have academic merit?

Yes

Is the work clearly and accurately presented and does it cite the current literature?

Partly

If applicable, is the statistical analysis and its interpretation appropriate?

Not applicable

Are all the source data underlying the results available to ensure full reproducibility?

Partly

Are the conclusions drawn adequately supported by the results?

Yes

Are sufficient details of methods and analysis provided to allow replication by others?

Partly

Reviewer Expertise:

Pyrometallurgy, process design.

I confirm that I have read this submission and believe that I have an appropriate level of expertise to confirm that it is of an acceptable scientific standard.

## References

[1] : Continuous scrap melting in a short rotary furnace. *Minerals Engineering* .2008;21(2) : 10.1016/j.mineng.2007.09.009 178-189 10.1016/j.mineng.2007.09.009

# Reviewer report: Reviewer response for version 2

**DOI:** 10.21956/openreseurope.22542.r56468

**Published:** 2025-08-29

**Authors:** Laura Donato

**Affiliations:** 1 Universite Libre de Bruxelles, Brussels, Brussels, Belgium

The authors have improved the manuscript in this revised version. The comments and suggestions from the previous review round have been carefully addressed, and the paper is now clear, well-structured, and scientifically sound. The methodology is appropriate and well-explained, the results are coherent and well-presented, and the conclusions are supported by the findings.

 I have no further major concerns. I recommend **acceptance** of this version for publication.

Is the study design appropriate and does the work have academic merit?

Partly

Is the work clearly and accurately presented and does it cite the current literature?

Partly

If applicable, is the statistical analysis and its interpretation appropriate?

Partly

Are all the source data underlying the results available to ensure full reproducibility?

Partly

Are the conclusions drawn adequately supported by the results?

Partly

Are sufficient details of methods and analysis provided to allow replication by others?

Partly

Reviewer Expertise:

Modelling Combustion systems, Machine Learning, Chemical Engineering

I confirm that I have read this submission and believe that I have an appropriate level of expertise to confirm that it is of an acceptable scientific standard.

# Reviewer report: Reviewer response for version 2

**DOI:** 10.21956/openreseurope.22542.r56469

**Published:** 2025-08-20

**Authors:** Neeraj Kumar Bhoi

**Affiliations:** 1 Mechatronics Engineering, Banasthali University (Ringgold ID: 29803), Jaipur, Rajasthan, India

Accept

Is the study design appropriate and does the work have academic merit?

Partly

Is the work clearly and accurately presented and does it cite the current literature?

Partly

If applicable, is the statistical analysis and its interpretation appropriate?

Partly

Are all the source data underlying the results available to ensure full reproducibility?

Partly

Are the conclusions drawn adequately supported by the results?

No

Are sufficient details of methods and analysis provided to allow replication by others?

Partly

Reviewer Expertise:

Sustainable manufacturing and finite element modeling study.

I confirm that I have read this submission and believe that I have an appropriate level of expertise to confirm that it is of an acceptable scientific standard.

# Reviewer report: Reviewer response for version 1

**DOI:** 10.21956/openreseurope.20114.r49082

**Published:** 2025-01-23

**Authors:** Shibo Kuang

**Affiliations:** 1 Monash University, Clayton, Victoria, Australia

This manuscript presents a numerical study of a top-blown rotary converter under specific conditions. However, the paper is poorly presented, and the results are unconvincing. Several major issues are outlined below:

1. The numerical model is not adequately justified, and key details are missing. It is unclear whether the interfaces among gas, metal, and slag were modeled, and, if so, how this was achieved. Additionally, the coupling between flows and heat transfer, as well as the interactions among different phases, are not clearly explained. Furthermore, the approach used for modeling solidification is entirely omitted, leaving significant methodological gaps.
2. The model has not been validated, which undermines the credibility of the results. Without validation against experimental data or established benchmarks, the reliability and accuracy of the study cannot be assessed.
3. The practical applicability of the results is unclear. The manuscript does not explain how the findings could be used to solve problems or improve the operation of top-blown rotary converters, making their relevance questionable.
4. The study fails to provide adequate context regarding progress in the field. Existing research gaps are not clearly identified, and the contribution of this work to advancing the field is not demonstrated.
5. The analysis presented in the study lacks depth. Key findings are not explored in detail, and important insights are either overlooked or insufficiently addressed.
6. The conclusions are overly specific and do not offer broader insights or generalizations. They should be expanded to highlight the implications of the findings and their relevance to the wider research community.

Is the study design appropriate and does the work have academic merit?

No

Is the work clearly and accurately presented and does it cite the current literature?

No

If applicable, is the statistical analysis and its interpretation appropriate?

No

Are all the source data underlying the results available to ensure full reproducibility?

No source data required

Are the conclusions drawn adequately supported by the results?

No

Are sufficient details of methods and analysis provided to allow replication by others?

No

Reviewer Expertise:

Computational process engineering

I confirm that I have read this submission and believe that I have an appropriate level of expertise to state that I do not consider it to be of an acceptable scientific standard, for reasons outlined above.

# Response

**Published:** 2025-06-30

**Authors:** Sergey Semenov

**Affiliations:** SIMTEC, Grenoble, France

We are grateful to the reviewer for the useful and insightful comments, which helped us to improve key aspects of our research article. Our responses to the individual comments in the report are provided below.

 1. The numerical model is not adequately justified, and key details are missing. It is unclear whether the interfaces among gas, metal, and slag were modeled, and, if so, how this was achieved. Additionally, the coupling between flows and heat transfer, as well as the interactions among different phases, are not clearly explained. Furthermore, the approach used for modeling solidification is entirely omitted, leaving significant methodological gaps.

**Authors: **We appreciate the reviewer’s feedback and would like to clarify the aspects raised regarding the justification and details of our numerical model. The boundary conditions, formulated as mathematical equations, define the exchange of momentum and energy at the interfaces and fully describe our approach to modeling the interfaces among gas, metal, and slag. These equations are explicitly presented in Section 4. To further enhance clarity, we have added additional explanations in Section 4 to explicitly outline how these interfaces are treated in our model. In particular: “The interfaces among gas, metal and slag are assumed to be fixed in space. The free surface displacement is disregarded, since the stratification of the phases in the Earth’s gravitational field is assumed to prevent significant interfaces displacement.” The coupling between flow and heat transfer is explicitly addressed by incorporating the influence of both flow velocity and turbulent thermal conductivity on the heat transfer process. Specifically, convective heat transfer is driven by the fluid motion, with velocity fields influencing temperature distribution. Additionally, the turbulence model accounts for enhanced thermal transport by incorporating turbulent thermal conductivity, which plays a key role in energy exchange within the system. These aspects are carefully described in Section 4.3, providing a comprehensive explanation of the coupling mechanisms implemented in the model. Additionally, the properties of metal and slag, including density and dynamic viscosity, are treated as functions of temperature, creating a strong bidirectional coupling between flow and heat transfer. These aspects of the model are clarified in sections 4 and 5 of the revised article. Regarding the modeling of solidification, our approach is explicitly described at the beginning of Section 4.3. Specifically, we employ the Apparent Heat Capacity method. This methodology effectively captures the phase transition process and is consistent with established modeling approaches in the field. We hope these clarifications address the reviewer’s concerns and demonstrate the completeness of our numerical model. We remain open to providing further elaboration if needed.

 2. The model has not been validated, which undermines the credibility of the results. Without validation against experimental data or established benchmarks, the reliability and accuracy of the study cannot be assessed. 

**Authors: **We appreciate the reviewer's concern about model validation. We acknowledge that experimental validation is a crucial step in establishing the accuracy of modelling approaches. However, experimental validation was beyond the scope of the current project. The primary objective of this study was to develop and explore a computational framework to numerically validate the new TBRC design and to gain insight into its optimal operation prior to its construction. This assumes the non-existence of the equipment and the absence of experimental data during the modelling work. A posteriori, when the new equipment was built, it was not possible to collect sufficient experimental data for the model validation, because the real operating conditions were not the same as in the model (for example, the TBRC lid was used in real operating conditions, as is recommended in this article). We acknowledge that experimental validation or comparison with established benchmarks would further strengthen our findings. We consider this to be an important direction for future work and encourage experimental studies to help validate our predictions (the appropriate statement is added to the conclusions).

 3. The practical applicability of the results is unclear. The manuscript does not explain how the findings could be used to solve problems or improve the operation of top-blown rotary converters, making their relevance questionable.

**Authors: **We would like to clarify that the study was carried out with a focus on a specific experimental Top-Blown Rotary Converter (TBRC) unit being designed and constructed at Mintek (South Africa). The objective was to analyze and optimize certain operational aspects - such as burner power, rotation frequency and inclination angle - within the context of that unit. While the scope of the study was limited to this particular TBRC, the methodology and findings offer broader insights that can be adapted to similar converter systems. For example, the modeling approach can be used by engineers to assess and enhance the performance of their own converters, especially under comparable conditions. To address this more clearly, we have revised the manuscript to include a discussion of how the results can inform decision-making and process improvements in other TBRC operations (see the end of section 6).

 4. The study fails to provide adequate context regarding progress in the field. Existing research gaps are not clearly identified, and the contribution of this work to advancing the field is not demonstrated. 

**Authors: **We appreciate the reviewer’s comment and understand the importance of clearly situating the study within the broader context of the field. In this work, our focus was on addressing a specific engineering challenge within the SisAl Pilot project, rather than providing a comprehensive review of the field. Nevertheless, we have revised the introduction to better highlight relevant prior work, clarify existing research gaps, and more explicitly articulate the contribution of our study within this targeted context.

 5. The analysis presented in the study lacks depth. Key findings are not explored in detail, and important insights are either overlooked or insufficiently addressed. 

**Authors: **We thank the reviewer for their thoughtful comment. While we agree that a more in-depth analysis could provide additional insights, we respectfully note that the primary objective of this numerical study is to validate a specific TBRC design by verifying its key operational aspects through simulations prior to construction, thereby ensuring successful commissioning and reliable operation thereafter. While further in-depth analysis of the findings is possible, it is not required for the purposes of the SisAl project and is therefore beyond the scope of this study. In the revised manuscript we have listed key findings at the end of section 6.

 6. The conclusions are overly specific and do not offer broader insights or generalizations. They should be expanded to highlight the implications of the findings and their relevance to the wider research community. 

**Authors: **We thank the reviewer for their helpful comment. In the revised manuscript, the broader-level findings are listed at the end of section 6 and are also summarized in the conclusions. We hope that this modification addresses the concerns of the reviewer.

# Reviewer report: Reviewer response for version 1

**DOI:** 10.21956/openreseurope.20114.r46861

**Published:** 2025-01-10

**Authors:** Laura Donato

**Affiliations:** 1 Universite Libre de Bruxelles, Brussels, Brussels, Belgium

This study evaluates the aluminothermic reduction of silica in a top-blown rotary converter (TBRC) heated by an oxy-fuel burner, offering low CO₂ emissions compared to carbothermic methods. Modeling focuses on the TBRC's thermal performance, assessing slag solidification risks, optimal burner power, and the effects of inclination and rotation of the TBRC. The software used for this purpose is COMSOL Multiphysics.

**Strengths**: 

 The research supports sustainable silicon production by promoting carbon-efficient technologies, in line with environmental goals. I found the paper interesting and consider it a strong starting point for sustainable silicon production. The geometry of the TBRC is well-defined, and the clarity of the methodology is evident throughout the work. Many of the figures are well-crafted and meaningful. However, I would have appreciated including a real-life image of the TBRC to enhance the understanding of the setup further. Overall, the paper provides valuable insights and a clear contribution to the field. 

**Drawbacks**: 

 To improve this study, I propose the following:

 - Literature Review: a more in-depth literature review on this type of research is necessary. The study does not reference similar works or discussions on this topic. Highlighting differences and strengths compared to existing studies would provide valuable outcomes and enhance the impact of the work, especially in the sustainability scenario. 

 - Chemical Reactions: Including the chemistry behind aluminothermic reduction and discussing its comparison with carbothermic would enrich the study. A detailed explanation of the processes could also help underline the significance of the proposed approach.

 - Experimental Data: Adding experimental data would significantly strengthen the study’s relevance and application, though I understand it may be challenging to incorporate at this stage.

**Additional comments:**

 - Was the computational mesh developed symmetrically? Do the 27,600 finite elements represent only half of the entire TBRC?

 - Could Figure 12 be made more comprehensive? I believe the labeling could be improved for better clarity.

 - Section 6.3: Why was the aluminothermic reduction assumed to last 20 minutes? Is there a reference or experimental data that justifies this duration?

Is the study design appropriate and does the work have academic merit?

Partly

Is the work clearly and accurately presented and does it cite the current literature?

Partly

If applicable, is the statistical analysis and its interpretation appropriate?

Partly

Are all the source data underlying the results available to ensure full reproducibility?

Partly

Are the conclusions drawn adequately supported by the results?

Partly

Are sufficient details of methods and analysis provided to allow replication by others?

Partly

Reviewer Expertise:

Modelling Combustion systems, Machine Learning, Chemical Engineering

I confirm that I have read this submission and believe that I have an appropriate level of expertise to confirm that it is of an acceptable scientific standard, however I have significant reservations, as outlined above.

# Response

**Published:** 2025-06-30

**Authors:** Sergey Semenov

**Affiliations:** SIMTEC, Grenoble, France

We are grateful to the reviewer for the useful and insightful comments, which helped us to improve key aspects of our research article. Our responses to the individual comments in the report are provided below.

 - Literature Review: a more in-depth literature review on this type of research is necessary. The study does not reference similar works or discussions on this topic. Highlighting differences and strengths compared to existing studies would provide valuable outcomes and enhance the impact of the work, especially in the sustainability scenario. 

**Authors: **Thank you for your valuable feedback. Additional literature sources on the sustainability and economic viability of the SisAl process, as well as on numerical modelling of rotary furnaces are cited and shortly discussed in the article introduction. Description of differences in thermal boundary conditions compared to a recent literature source is mentioned in section 4.3. Another key distinguishing aspect of our work compared to previous studies is the problem geometry and operating conditions, which directly influence the flow dynamics and heat transfer.

 - Chemical Reactions: Including the chemistry behind aluminothermic reduction and discussing its comparison with carbothermic would enrich the study. A detailed explanation of the processes could also help underline the significance of the proposed approach.

**Authors: **The revised article introduction describes the chemical reactions of the traditional carbothermic reduction of silica and the aluminothermic reduction proposed in the SisAl Pilot project. The advantages of the SisAl process over the carbothermic process are explained in detail, justifying its significance and economic viability.

 - Experimental Data: Adding experimental data would significantly strengthen the study’s relevance and application, though I understand it may be challenging to incorporate at this stage.

**Authors: **We appreciate the reviewer’s insightful comment on the need for experimental validation to support our numerical results. However, experimental validation was beyond the scope of this study. The whole purpose of this work is to numerically validate the new TBRC design and to gain insight into its optimal operation prior to its construction. This assumes the non-existence of the equipment and the absence of experimental data during the modelling work. A posteriori, when the new equipment was built, it was not possible to collect sufficient experimental data for the model validation, because the real operating conditions were not the same as in the model (for example, the TBRC lid was used in real operating conditions, as is recommended in this article). We acknowledge that experimental validation or comparison with established benchmarks would further strengthen our findings. We consider this to be an important direction for future work and encourage experimental studies to help validate our predictions (the appropriate statement is added to the conclusions).

**Additional comments:**

 - Was the computational mesh developed symmetrically? Do the 27,600 finite elements represent only half of the entire TBRC?

**Authors: **Since the computation is done in a 2D axisymmetric geometry, the computational domain (see Figure 3) represents only half of the entire TBRC in the r-z plane. The other half of TBRC does not exist in a 2D axisymmetric formulation and therefore cannot be meshed. Thus, we can say that 27600 finite elements represent only half of the entire TBRC.

 - Could Figure 12 be made more comprehensive? I believe the labeling could be improved for better clarity.

**Authors: **Thank you for this suggestion. The labeling of Figure 12 is improved in the revised article.

 - Section 6.3: Why was the aluminothermic reduction assumed to last 20 minutes? Is there a reference or experimental data that justifies this duration?

**Authors: **We appreciate the reviewer's valuable comment on the need for justification, which helps us to improve the quality of our work. The aluminothermic reduction is assumed to last for 20 minutes based on experimental investigations carried out in the framework of the SisAl Pilot project. A detailed explanation with a literature source is given at the beginning of section 6.3 of the revised article.

# Reviewer report: Reviewer response for version 1

**DOI:** 10.21956/openreseurope.20114.r46858

**Published:** 2024-11-28

**Authors:** Neeraj Kumar Bhoi

**Affiliations:** 1 Mechatronics Engineering, Banasthali University (Ringgold ID: 29803), Jaipur, Rajasthan, India

**Strengths:**

1. The study provides a comprehensive numerical model for analyzing preheating and charge heating in a top-blown rotary converter (TBRC) using COMSOL Multiphysics.
2. The use of the finite element method and detailed thermal and fluid dynamics modeling ensures accuracy and relevance.
3. The research contributes to sustainable silicon production by addressing carbon-emission-friendly technologies, aligning with global environmental priorities.
4. Clear presentation of results, including the influence of burner power, TBRC inclination angle, and rotation frequency, with practical recommendations to improve operational efficiency.
5. The creation of a decision-making tool adds value for real-world applications and pilot projects.

**Areas for Improvement:**

1. **Experimental Validation:** The paper relies entirely on numerical modeling. Incorporating experimental validation of the model's predictions would enhance credibility and applicability to real-world scenarios.
2. **Data Uncertainty:** Addressing uncertainties in material properties, boundary conditions, and numerical methods could improve reliability. Sensitivity analysis of key parameters would add depth.
3. **Generalization of Results:** The study focuses on specific configurations and conditions. Broadening the scope to include different TBRC designs and operational scenarios could increase its relevance.
4. **Economic Viability:** While the paper addresses technical aspects, incorporating a discussion on the economic feasibility of the proposed configurations would strengthen its industrial impact.
5. **Environmental Assessment:** A more detailed assessment of the environmental benefits, such as a reduction in CO2 emissions compared to traditional methods, would align better with sustainability goals.
6. **Visualization:** Some figures (e.g., temperature fields and slag behavior) could benefit from clearer annotations and labels to aid interpretation.
7. **Policy Implications:** Linking the findings to broader industrial standards or regulatory frameworks could highlight the significance of the work for policymakers.
8. **References to Prior Work:** The paper could discuss how this work builds on or differs from existing studies on TBRC and aluminothermic reduction, particularly addressing gaps in previous literature.

Is the study design appropriate and does the work have academic merit?

Partly

Is the work clearly and accurately presented and does it cite the current literature?

Partly

If applicable, is the statistical analysis and its interpretation appropriate?

Partly

Are all the source data underlying the results available to ensure full reproducibility?

Partly

Are the conclusions drawn adequately supported by the results?

No

Are sufficient details of methods and analysis provided to allow replication by others?

Partly

Reviewer Expertise:

Sustainable manufacturing and finite element modeling study.

I confirm that I have read this submission and believe that I have an appropriate level of expertise to confirm that it is of an acceptable scientific standard, however I have significant reservations, as outlined above.

# Response

**Published:** 2025-06-27

**Authors:** Sergey Semenov

**Affiliations:** SIMTEC, Grenoble, France

We are grateful to the reviewer for the useful and insightful comments, which helped us to improve key aspects of our research article. Our responses to the individual comments in the report are provided below.

**1. Experimental Validation:** The paper relies entirely on numerical modeling. Incorporating experimental validation of the model's predictions would enhance credibility and applicability to real-world scenarios.

**Authors: **We appreciate the reviewer’s insightful comment on the need for experimental validation to support our numerical results. However, experimental validation was beyond the scope of this study. The whole purpose of this work is to numerically validate the new TBRC design and to gain insight into its optimal operation prior to its construction. This assumes the non-existence of the equipment and the absence of experimental data during the modelling work. A posteriori, when the new equipment was built, it was not possible to collect sufficient experimental data for the model validation, because the real operating conditions were not the same as in the model (for example, the TBRC lid was used in real operating conditions, as is recommended in this article). We acknowledge that experimental validation or comparison with established benchmarks would further strengthen our findings. We consider this to be an important direction for future work and encourage experimental studies to help validate our predictions (the appropriate statement is added to the conclusions).

**2. Data Uncertainty:** Addressing uncertainties in material properties, boundary conditions, and numerical methods could improve reliability. Sensitivity analysis of key parameters would add depth.

**Authors: **We appreciate the reviewer’s valuable suggestion regarding the inclusion of additional parameters in the parametric study. We fully acknowledge the importance of exploring the sensitivity to material properties, numerical methods, and boundary conditions; however, extending the study to incorporate these factors would significantly increase the computational cost. Our current analysis already involves a parametric study on three key parameters (burner power, inclination angle and rotation frequency), leading to a three-dimensional parameter space. Introducing additional parameters would exponentially increase the number of simulations required, making the problem computationally prohibitive within the available resources. The numerical methods and boundary conditions used in this work are consistent with established best practice for modelling rotary converters, see for example [Crego et al. Transient mathematical modelling of a gas rotary furnace for melting slag. Applied Thermal Engineering. 2024;246:122928].

**3. Generalization of Results:** The study focuses on specific configurations and conditions. Broadening the scope to include different TBRC designs and operational scenarios could increase its relevance.

**Authors: **We appreciate the reviewer’s suggestion regarding the broadening of the study to encompass additional designs and operational scenarios. We fully acknowledge that a more extensive exploration would enhance the general applicability of our findings. However, our study aims to provide an in-depth analysis of a well-defined configuration that is representative of practical applications. Expanding the scope significantly would require a substantial increase in computational resources and analysis time, which is beyond the scope of current study. To ensure the robustness of our conclusions, we have selected the studied configuration based on its practical relevance and have conducted parametric studies to capture key trends. We believe that the insights gained from this work can serve as a foundation for future studies that may explore broader design variations and operating conditions. We hope this clarification addresses the reviewer’s concern.

**4. Economic Viability:** While the paper addresses technical aspects, incorporating a discussion on the economic feasibility of the proposed configurations would strengthen its industrial impact.

**Authors: **We fully acknowledge that assessing economic viability is crucial for industrial applications. However, this study is primarily focused on the technical and physical aspects of the proposed configurations, with an emphasis on performance evaluation. Evaluating the economic feasibility for the specific configuration of the metallurgical reactor studied in this article is challenging, as the technology implemented in the studied process differs from the originally planned approach. For example, to mitigate the impact of load shedding in South Africa, electrical heating was replaced with a gas burner. It is important to note that this research is part of a larger collaborative effort involving multiple partners, and the economic feasibility of the overall SisAl process is assessed at a broader level. See for example the following reference [H. Philipson et al., Preliminary techno-economic considerations of the SisAl process-closing materials loops through industrial symbiosis. *Proceedings of the Silicon for the Chemical & Solar Industry XVI*], which is cited in the revised version of the article.

**5. Environmental Assessment:** A more detailed assessment of the environmental benefits, such as a reduction in CO2 emissions compared to traditional methods, would align better with sustainability goals.

**Authors: **As previously mentioned, the use of a gas burner is a mitigation measure that alters the CO₂ impact. The originally planned technology, which relied on electrical heating and aluminothermic reduction, would have resulted in nearly zero CO₂ emissions, except for those associated with electricity production. In this context, evaluating the environmental impact of a specific technological step that includes mitigation measures is not considered relevant.

**6. Visualization:** Some figures (e.g., temperature fields and slag behavior) could benefit from clearer annotations and labels to aid interpretation.

**Authors: **Figures 4, 7, 8, 12, 13, 15 are modified. Additional labels are added for temperature values and slag behavior.

**7. Policy Implications:** Linking the findings to broader industrial standards or regulatory frameworks could highlight the significance of the work for policymakers.

**Authors: **We fully acknowledge that aligning research with established guidelines enhances its impact, particularly for policymakers and industry stakeholders. The primary focus of this study has been on the technical and scientific aspects of the process, and an in-depth regulatory analysis was beyond its intended scope. Future work could explore this aspect in greater detail.

**8. References to Prior Work:** The paper could discuss how this work builds on or differs from existing studies on TBRC and aluminothermic reduction, particularly addressing gaps in previous literature.

**Authors: **Additional literature sources on the sustainability and economic viability of the SisAl process, as well as on numerical modelling of rotary furnaces are cited and shortly discussed in the article introduction. Description of differences in thermal boundary conditions compared to a recent literature source is mentioned in section 4.3. Another key distinguishing aspect of our work compared to previous studies is the problem geometry and operating conditions, which directly influence the flow dynamics and heat transfer. We have now clarified this point in the introduction of the revised article.
